# Perovskite seeding growth of formamidinium-lead-iodide-based perovskites for efficient and stable solar cells

**DOI:** 10.1038/s41467-018-04029-7

**Published:** 2018-04-23

**Authors:** Yicheng Zhao, Hairen Tan, Haifeng Yuan, Zhenyu Yang, James Z. Fan, Junghwan Kim, Oleksandr Voznyy, Xiwen Gong, Li Na Quan, Chih Shan Tan, Johan Hofkens, Dapeng Yu, Qing Zhao, Edward H. Sargent

**Affiliations:** 1 Department of Electrical and Computer Engineering, University of Toronto, 35 St. George Street, Toronto, ON M5S 1A4 Canada; 2 State Key Laboratory for Mesoscopic Physics and Electron Microscopy Laboratory, School of Physics, Peking University, 100871 Beijing, China; 3 Department of Chemistry, KU Leuven, Celestijnenlaan 200F, B-3001 Leuven, Belgium; 4 Collaborative Innovation Center of Quantum Matter, 100084 Beijing, China

## Abstract

Formamidinium-lead-iodide (FAPbI_3_)-based perovskites with bandgap below 1.55 eV are of interest for photovoltaics in view of their close-to-ideal bandgap. Record-performance FAPbI_3_-based solar cells have relied on fabrication via the sequential-deposition method; however, these devices exhibit unstable output under illumination due to the difficulty of incorporating cesium cations (stabilizer) in sequentially deposited films. Here we devise a perovskite seeding method that efficiently incorporates cesium and beneficially modulates perovskite crystallization. First, perovskite seed crystals are embedded in the PbI_2_ film. The perovskite seeds serve as cesium sources and act as nuclei to facilitate crystallization during the formation of perovskite. Perovskite films with perovskite seeding growth exhibit a lowered trap density, and the resulting planar solar cells achieve stabilized efficiency of 21.5% with a high open-circuit voltage of 1.13 V and a fill factor that exceeds 80%. The Cs-containing FAPbI_3_-based devices show a striking improvement in operational stability and retain 60% of their initial efficiency after 140 h operation under one sun illumination.

## Abstract (web summary)

Formamidinium-lead-iodide-based perovskites have a preferred bandgap below 1.55 eV for solar cell applications but suffer from operational instability. Here, Zhao et al. improve the film quality using cesium-containing seeded growth to show high stabilized efficiency and more than 100 h lifetime under simulated sunlight.

## Introduction

Hybrid perovskite solar cells have been developed for efficient solar energy conversion in light of the devices' high power conversion efficiencies (PCEs) and facile processing^1–6^. Processing techniques such as one-step antisolvent crystallization and two-step sequential deposition methods have been developed to produce high-quality perovskite thin films^7–10^. The perovskite composition is tuned via cation and halide selection to manipulate the bandgap, stability, and transport properties of polycrystalline perovskite films^6,11–17^. Among the broad range of compositions, formamidinium-lead-iodide (FAPbI_3_)-based perovskites with minimal bromide anions and bandgap below 1.55 eV, namely (FAPbI_3_)_1-*x*_(MAPbBr_3_)_*x*_ where *x* is less than 0.05 (hereafter referred to as FAPbI_3_-based perovskites), are of intense interest for photovoltaic applications: they possess a close-to-ideal bandgap and a certified record PCE of 22.7%^2,3,10,12,18–21^.

FAPbI_3_-based perovskite solar cells have demonstrated superior initial performance and dark storage stability; however, the devices exhibit rapid performance degradation following operation at their maximum power point (MPP) even for a few hours under illumination^10,19,22,23^. Their operational stabilty is far inferior to mixed cation-halide perovskites cells with high Bromide content (≥15%) via Cs or Rb incorporation (e.g., Cs-doped (FAPbI_3_)_0.85_(MAPbBr_3_)_0.15_ and FA_0.83_Cs_0.17_Pb(I_0.83_Br_0.17_)_3_)^6,14–17,20,24–27^. These studies demonstrate the crucial importance of the Cs cation additive for the long-term photostability of mixed cation-halide perovskites.

So far, only via two-step sequential deposition processing have superior-performing FAPbI_3_-based perovskite solar cells been achieved^2,3,10,19^. The lack of Cs cation in the sequentially deposited perovskite films is one important reason for the poor operational stability in previously reported FAPbI_3_-based photovoltaic devices^2,3,10,19,20,22^. In conventional two-step method, efficient incorporation of Cs in FAPbI_3_-based perovskites is curtailed by the low solubility of inorganic cesium-halide salts (CsX, where X = I, Br, Cl) in alcohols (typically isopropanol, IPA) and the limited diffusion depth of inorganic cations into PbI_2_ films^28^. Directly adding CsX in the PbI_2_ layer as the first step leads to the formation of the non-perovskite δ-phase CsPbI_3_^29,30^, and this leads to a failure to produce efficient photovoltaic devices^22^.

In addition to the deficiency of cesium incorporation, perovskite nucleation is poorly controlled in conventional two-step sequential processing: variability in the interdiffusion reaction between PbI_2_ and the organic compounds produces a substantial variation in device performance among processing batches^8,28,31^.

To reduce the gap between solar cell efficiency and operational stability, here we devise a perovskite seeding growth (PSG) method that leads to Cs-containing, high-quality FAPbI_3_-based perovskite films with a bandgap below 1.55 eV. This advance enables a significant improvement in device operational stability, device performance, and processing reproducibility of FAPbI_3_-based perovskite solar cells. Inspired by the seed-assisted growth of Si and GaAs semiconductor ingots^32–34^, we incorporate submicron-sized crystalline perovskite seeds in the PbI_2_ layer in the two-step sequential deposition. The perovskite seeds act as nuclei for the ensuing perovskite growth when alkylammonium halides react with PbI_2_. We successfully incorporate inorganic cations such as Cs into high-quality perovskite films using Cs-containing perovskite seeds. As a result, we achieve large grain sizes, low trap density, and quasi-single crystallographic orientation in perovskite films via the PSG method. Planar perovskite solar cells processed using the PSG method exhibit a high stabilized PCE of 21.5% and narrow batch-to-batch variation (1.4% absolute PCE). The Cs-containing FAPbI_3_-based perovskite solar cells exhibit a striking improvement in photostability and thermal stability over their non-Cs counterparts and retain over 60% of their initial stabilized efficiency after 140 h operation at MPP under one sun illumination. To the best of our knowledge, this represents the highest operational stability among FAPbI_3_-based perovskite solar cells with bandgaps below 1.55 eV^10,19,22,23,35–37^.

## Results

### Fabrication of perovskite thin film via PSG

**Fig. 1:**
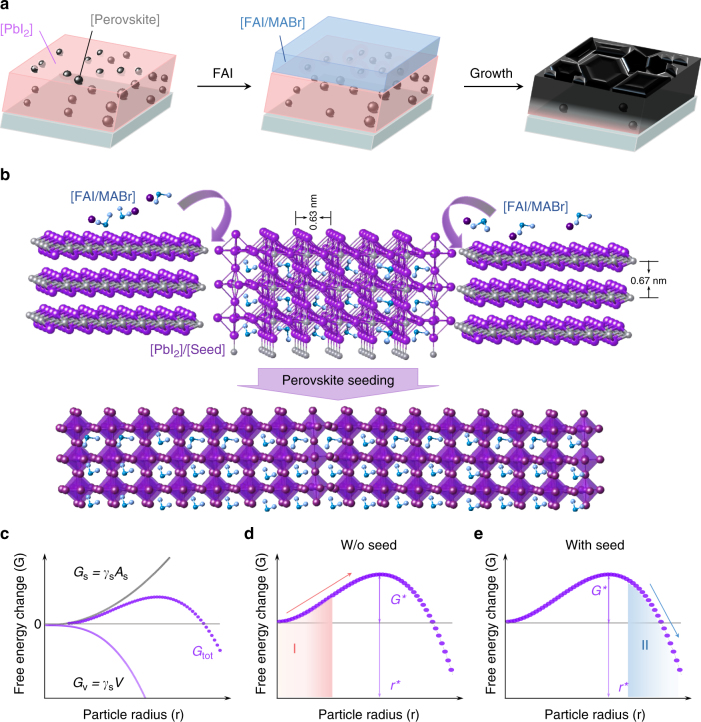
Perovskite thin films prepared by perovskite seeding growth. **a** Schematic of fabrication procedure of perovskite films using perovskite seeding growth. **b** Schematic showing PbI_2_ transformation to perovskite starting from perovskite seeds due to the lower energy barrier to perovskite crystallization. **c** The Gibbs energy *G*_tot_ as a function of particle radius. *G*_tot_ consists of a volume term *G*_v_ and a surface term *G*_s_, where *γ*_s_ and *γ*_v_ are the surface free energy per unit area and volume free energy per unit volume, respectively; **d**–**e** Evolution of *G*_tot_ during the crystallization process without and with the seed crystal, respectively. In the absence of seed crystal, a nucleus must first be formed, and the nucleus must then overcome the energy barrier G^*^ to grow beyond the critical radius r^*^ (Region I). In the case of perovskite seeds embedded in PbI_2_, the size of the seed crystal is already larger than r^*^ and thus the crystallization commences spontaneously from the seed (Region II)

The fabrication of FAPbI_3_-based perovskite films via the PSG method is depicted in Fig. 1a and b. We first prepare a PbI_2_ layer that contains submicron-sized perovskite seeds with the composition Cs_0.10_FA_0.78_MA_0.12_PbI_2.55_Br_0.45_ (we abbreviate to Cs_0.1_MAFA). Atop the PbI_2_ layer, we deposit the mixed organic halide salts FAI/MABr/MACl (0.425/0.065/0.113 M in IPA). As shown in Fig. 1b, the perovskite seeds act as nuclei that facilitate perovskite formation during the diffusion of the organic compounds into the PbI_2_ layer.

The kinetics of the crystal growth process can be explained through a Gibbs free energy that includes a volume term (*G*_v_) and a surface term (*G*_s_)^8^. Once a new nucleus forms (particle size larger than critical radius r^*^), the crystal grows spontaneously during the ensuing reaction process; otherwise, it will disintegrate spontaneously (Fig. 1c). In the conventional two-step method, the crystallization of perovskites will not occur until the formation of nuclei, for which a critical free energy G^*^ has to be overcome^2,3,8–10,38^ as depicted in region I of Fig. 1d. In the PSG method, perovskite growth commences immediately from the perovskite seeds (nucleation centers) when the alkylammonium halide salts are deposited on the PbI_2_ film (region II Fig. 1e). In addition, perovskite seeds facilitate the diffusion and intercalation of organic cations and halide anions into the layered PbI_2_ via the PbI_2_/perovskite interface channels^3,9,39^. This is manifested in a faster perovskite crystallization process along the edges of PbI_2_ single crystal (Supplementary Fig. 1).

**Fig. 2:**
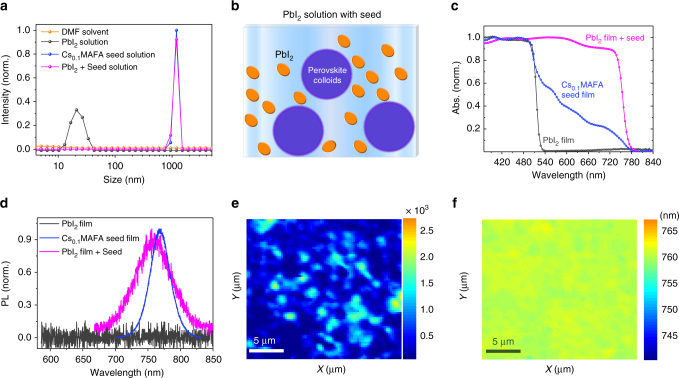
Optical characterization of the PbI_2_ film with perovskite seeds. **a** Dynamic light scattering spectra of the pure PbI_2_ solution (0.00028 M), pure perovskite precursor solution (0.00028 M), and the PbI_2_ solution mixed with perovskite precursor solution (0.00028 M for PbI_2_ and 0.00004 M for perovskite precursor). The size indicates the colloidal particle size in solution. **b** Illustration of the colloids of perovskite precursor in PbI_2_ solution. **c**, **d** The absorption and PL spectra of pure PbI_2_ film, perovskite seed film with a composition of Cs_0.10_FA_0.78_MA_0.12_PbI_2.55_Br_0.45_, and PbI_2_ film with perovskite seeds (seed concentration 14 vol.%). **e–f** Confocal PL mapping of the intensity (**e**) and peak position (**f**) on the PbI_2_ film with perovskite seeds

To fabricate PbI_2_ films containing submicron-sized perovskite seeds, we first prepared two separate solutions of PbI_2_ and perovskite precursor. It has been shown that perovskite precursors are dispersed as colloids in processing solvents^40–42^. Following addition of the perovskite precursor colloidal solution into the PbI_2_ solution, these colloids are well preserved in the PbI_2_ solution, as is evident from their dynamic light scattering and absorption profiles (Fig. 2a and Supplementary Fig. 2). A schematic of the solution is given in Fig. 2b. The perovskite seeds are formed in the PbI_2_ film by annealing the spin-coated film at 70 °C for 2 min. The formation of crystalline perovskite seeds is confirmed via absorption and photoluminescence (PL) spectra (Fig. 2c–d). The absorption spectrum of the PbI_2_ film with perovskite seeds presents two absorption plateaus at around 520 and 780 nm that originate from PbI_2_ and perovskite seeds, respectively^9^. The PL peak position and absorption edge of the PbI_2_ film with seeds are the same as that of Cs_0.1_MAFA perovskite seed films processed using the anti-solvent method. The crystalline perovskite seeds fail to form in the PbI_2_ film if we directly mix the powder-form precursors within the PbI_2_ solution (Supplementary Fig. 3). This highlights the importance of solvated colloids of perovskite precursor in the PSG method.

We applied confocal PL mapping on the sample prepared from PbI_2_ solution with 14 vol.% perovskite seeds to obtain the spatial distribution of seeds in the PbI_2_ film. Here the seed concentration is defined as the volume fraction of perovskite precursor solution added into the PbI_2_ solution. From the PL intensity maps (Fig. 2e), we see that the perovskite seeds (emissive spots) are well dispersed in the PbI_2_ film (non-luminescent background in deep blue). As indicated by the very similar PL peak positions over the mapped region (Fig. 2f), the perovskite seeds at different locations within the PbI_2_ film shall have an almost identical composition. The density of perovskite seeds in the PbI_2_ film increases with higher seed concentration in the PbI_2_ solution (Supplementary Fig. 4).

**Fig. 3:**
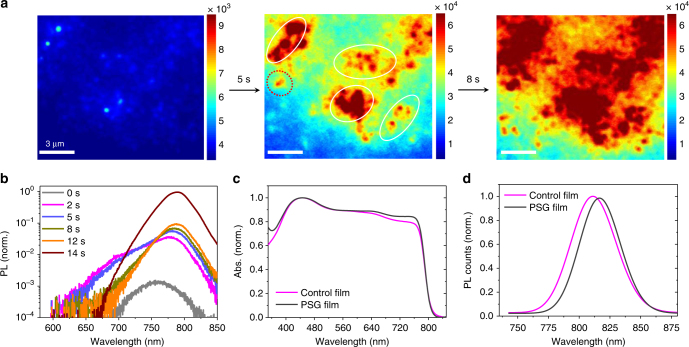
PL imaging of the growth of perovskite seeded films. **a** In situ photoluminescence microscopy reports real-time growth of perovskite from the pre-embedded perovskite seeds. The white circles indicate locations with perovskite-seed-assisted growth, while red dashed circles indicate locations with random nucleation process. The color bar indicates the PL intensity emitted from the sample. **b** The evolution of PL spectra of a perovskite-seeded region during the reaction process between PbI_2_ and the alkylammonium halides. Note that the substrate was kept at room temperature during the observation. **c**, **d** The absorption and PL spectra of the annealed perovskite films fabricated via conventional two-step sequential deposition (control film) and perovskite seeding growth (PSG film) with 14 vol.% seed concentration. The films were annealed at 140 °C for 25 min. The PL signal was collected from the back side of glass with an excitation wavelength at 540 nm

We then carried out in situ PL imaging to investigate the perovskite crystallization process upon the deposition of organic ammonium salts solution onto seed-containing PbI_2_ film. We did so by tracking the PL emission evolution of the perovskite phase. Figure 3a (left) shows the PL mapping of the perovskite-seed-PbI_2_ solid film before the deposition of the organic ammonium salts. The perovskite crystallization growth starts immediately from the seeds when the ammonium salt solution is placed on the seed-containing PbI_2_ film. After 2 s, two distinct types of growth kinetics can be observed (Fig. 3a, middle): one is perovskite-seed-assisted growth (white circles) and the other is random nucleation (red dashed circles) without the participation of seeds. The growth rate from the perovskite-seed sites is much faster than growth without the presence of seeds. The growth rate from the perovskite-seed sites reaches about 1 µm s^−1^. Overall, the perovskite-seed-assisted growth dominates the perovskite crystallization process across the entire duration of the reaction process (Fig. 3a, right). The time-dependent PL spectra from an area with perovskite seeds show a progressive red-shift in peak position and increased emission intensity with reaction time (Fig. 3b). This behavior indicates efficient ion exchange between the perovskite seeds and as-formed perovskite crystals during rapid perovskite growth^43,44^. We observe a substantially uniform distribution of elements in as-prepared perovskite film after annealing at 140 °C for 25 min, which is evidenced by 2-D element mapping via Time-Of-Flight Secondary Ion Mass Spectrometry (ToF-SIMS) (Supplementary Fig. 5).

### Characterization of perovskite films

Figure 3c and d presents optical characterization of the resulting perovskite films fabricated by the conventional two-step method vs. via the PSG method. We refer to the samples as control and PSG, respectively. Here we observe a similar absorption profile in both control and PSG films (Fig. 3c). Our FAPbI_3_-based perovskite films achieved here possess a similar absorption edge (815 nm) with previous reports^2,3,10^. The optical band gap almost remains the same (1.53 eV) for various seed concentrations from 0 to 24 vol.%. From the PL spectra, the full width at half maximum of PL in PSG sample (65 meV) is narrower than that in the control sample (88 meV), indicating reduced energy disorder in the PSG sample (Fig. 3d). The compositions of control sample and PSG sample (14 vol.% seed concentration) are estimated as (FAPbI_3_)_0.96_(MAPbBr_3_)_0.04_ and (Cs_0.02_FA_0.98_PbI_3_)_0.97_(MAPbBr_3_)_0.03_ from the X-ray photoelectron spectroscopy (Supplementary Fig. 6), where the Br contents are comparable to previous reports^2,3,10^. We further explored the chemical depth profiles of different ions in PSG samples via Ar^+^ etching, showing a higher ratio of Br at the top surface than the bottom of samples (Supplementary Fig. 7). This leads to different PL peak positions emitted from the surface and bottom of sequentially deposited perovskite film (Supplementary Fig. 8). The Cl is negligible in the resulting perovskite films (Supplementary Fig. 7), consistent with related previous studies^10^.

**Fig. 4:**
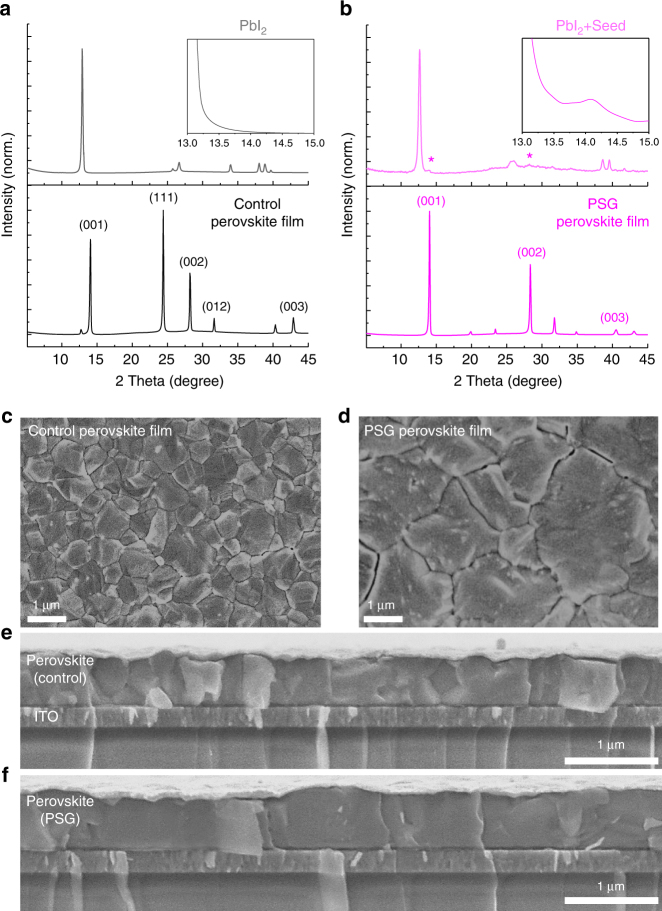
Structural and morphological characterization of perovskite films. **a** XRD patterns of pure PbI_2_ film and the as-prepared control perovskite film. The inset is a zoom-in of the XRD spectrum in the range 13°–15°. **b** XRD patterns of the PbI_2_ film with 14 vol.% perovskite seed and the as-prepared PSG perovskite film. The inset is a zoom-in of the XRD spectrum in the range 13^o^–15^o^. **c**–**d** Top-view SEM images of the control and PSG perovskite films. **e**,** f** Cross-sectional SEM images of the control and PSG films

We next compare the structure and morphology of the perovskite films using X-ray diffraction (XRD) and scanning electron microscopy (SEM). The PSG film has a preferential [001] orientation, while the control film has none as seen from the XRD patterns (Fig. 4a, b). The preferential orientation in PSG samples may result from that of the seeds in the PbI_2_ film that have the lowest interfacial energy. In contrast, there are no (orientable) seeds to modulate perovskite crystallization in the conventional two-step method (Fig. 4a, upper).

The SEM images show that the average grain size (3 µm^2^ in lateral area) in the PSG film is much larger than that of the control film (0.25 µm^2^) (Fig. 4c, d). We also compare the cross-sections of control and PSG films (around 700-nm thick). The PSG sample clearly shows a columnar character for the crystal grains from the bottom to top (Fig. 4e, f). The grain size is largely dependent on the nucleation density during the crystallizing process, and it depends as a result on perovskite-seed concentration^4,8^. The SEM images and XRD patterns of perovskite films with different seed concentrations are presented in Supplementary Fig. 9. A seed concentration higher than 14 vol.% leads to more random crystallographic orientation and smaller averaging grain size in the perovskite film. Furthermore, the lattice mismatch between seed and resulting perovskite affects the perovskite growth during the interdiffusion process of the organic compounds. The MAPbBr_3_ seed, which has a considerably smaller lattice constant than the target FAPbI_3_-based perovskite, leads to smaller grain size in the resulting perovskite film, and produces lower photovoltaic performance than that of the control sample (Supplementary Fig. 10).

### Photovoltaic devices and performance

To evaluate the quality of perovskite films produced by the PSG vs. the conventional two-step sequential method, we fabricated planar solar cells with the device structure ITO/TiO_2_-Cl/perovskite/Spiro-OMeTAD/Au^6^. By plotting the device efficiency vs. seed concentration (Supplementary Fig. 11), we observe an obvious increase of efficiency for seed concentrations below 20 vol.% compared to the control devices. We also investigated the effect of seed composition and found that Cs_0.1_MAFA is the best candidate among widely adopted perovskite compositions (Supplementary Fig. 11). The highest PCE is achieved for perovskite films fabricated from Cs_0.1_MAFA perovskite seeds with a seed concentration of 14 vol.%. Unless otherwise stated, this optimal condition is used for PSG samples in the text that followings.

**Fig. 5:**
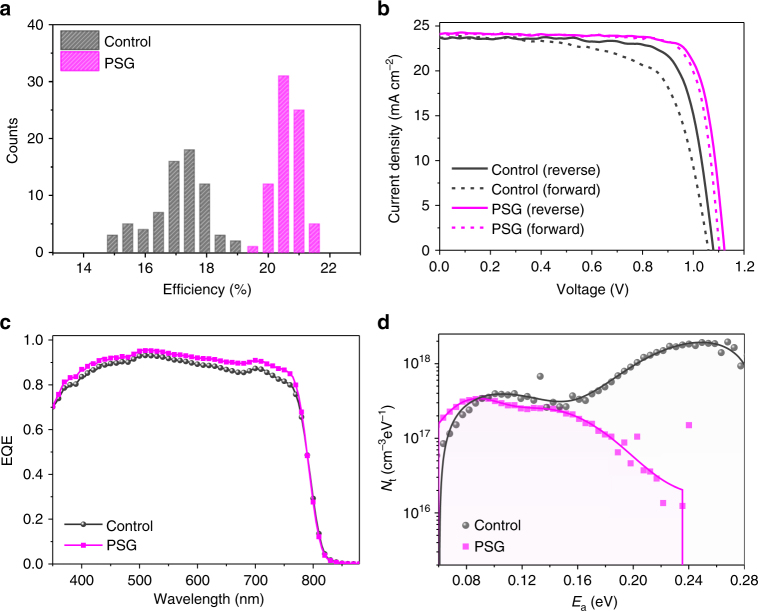
Photovoltaic performance of control and PSG perovskite solar cells. **a** Histogram of solar cell efficiencies for control and PSG devices. **b** *J*-*V* curves of the best-performing control and PSG devices with a scanning rate of 10 mV s^−1^ (voltage step of 10 mV and delay time of 1000 ms) from 1.15 to −0.01 V as the reverse scan, and from −0.01 to 1.15 V as the forward scan. **c** The external quantum efficiency (EQE) spectra of the control and PSG devices. **d** Trap density of states obtained by thermal admittance spectroscopy for control and PSG devices

The histogram of device PCEs for both control and PSG devices is shown in Fig. 5a. The PCE distribution of PSG devices is much narrower than that of control devices, with a significant drop in standard deviation from 3.2 to 1.4% (absolute PCE). The current density-voltage (*J*-*V*) curves for the best-performing control and PSG devices are shown in Fig. 5b. The open-circuit voltage (*V*_oc_) of the PSG device increases considerably by 60 mV and the efficiency increases from 19.4 (with *V*_oc_ = 1.07 V, *J*_sc_ = 23.7 mA cm^−2^, and FF = 0.75) to 21.7% (with *V*_oc_ = 1.13 V, *J*_sc_ = 24.1 mA cm^−2^, and FF = 0.81) for the reverse scans, where *J*_sc_ and FF are short-circuit current density and fill factor, respectively. The integrated *J*_sc_ values from the external quantum efficiency (EQE) spectra are 23.8 and 24.2 mA cm^−2^ for the control and PSG devices, respectively (Fig. 5c). The values are consistent with the *J*_sc_ values determined from *J*-*V* curves. The hysteresis in best PSG devices is negligible, with a small PCE discrepancy between forward and reverse scans (0.4% absolute PCE), whereas the control device exhibits a considerable hysteresis in *J*-*V* scans (Supplementary Table 1). The higher degree of hysteresis in control device is mainly due to the higher trap density in the perovskite film as we will discuss later^6,45,46^. One of our best PSG devices was sent to an accredited PV calibration center (National Institute of Metrology, China) for certification, which confirmed a PCE of 21.9% in reverse scan under AM 1.5G full-sun illumination with a scan rate of 40 mV s^−1^ (Supplementary Figs. 12–13).

To further investigate the performance improvement by the PSG method, we use thermal admittance spectroscopy to acquire the density of trap states in control and PSG devices^45,47,48^. The trap density distribution of the control device has two peaks at depth of 0.09 and 0.27 eV, corresponding to trap densities of 0.8 × 10^16^ cm^−3^ and 0.9 × 10^17^ cm^−3^, respectively. The PSG device shows a strikingly reduced trap density, especially for the deep-level defects. The PSG device shows a reduced trap-mediated recombination rate in impedance spectra (Supplementary Fig. 14), and this is accompanied by a longer PL decay lifetime (Supplementary Fig. 15) compared to the control device. We also fabricated electron-only and hole-only devices to study the origins of carrier trapping in perovskite films. SCLC reveals that electron traps are dominant in perovskite films (Supplementary Fig. 16)^2,49,50^. The PSG devices exhibit reduced electron traps, in agreement with their higher EQE values in the longer wavelength region as shown in Fig. 5c.

### Operational stability of perovskite solar cells

In addition to the high initial performance, solar cells must have stable output at MPP under constant illumination operating conditions^6,15,51^. An addition of a small amount of Cs cations has been previously shown to improve dramatically the photostability and thermal stability of high-performance mixed-cation, mixed-halide perovskite solar cells^6,14–17,20,36^ (Supplementary Table 2).

**Fig. 6:**
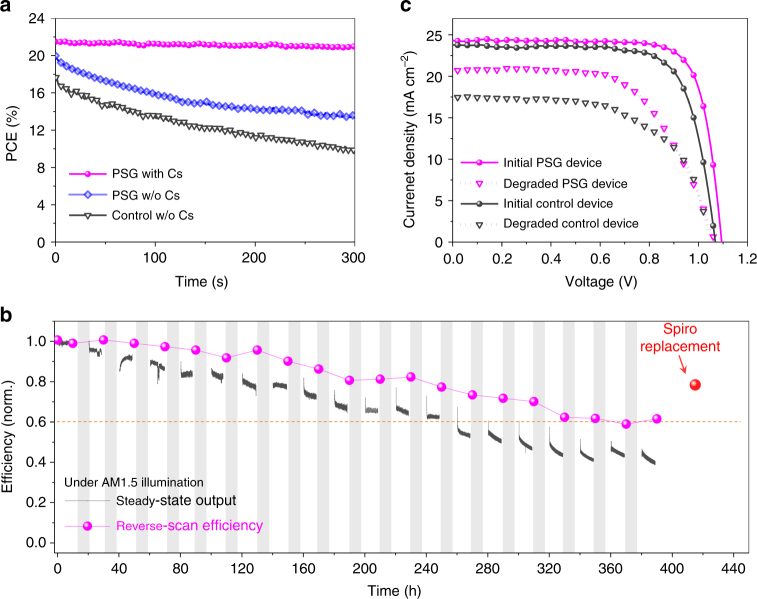
Stability characterization of perovskite solar cells. **a** The steady-state power output of the control device (w/o Cs) and PSG devices using MAFA (w/o Cs) and Cs_0.1_MAFA (with Cs) seeds measured at MPP under AM 1.5G full-sun illumination. **b** Long-term operational stability test for the PSG device under AM 1.5G illumination with a 420 nm cutoff UV filter. The device was repeatedly operated at MPP for 10 h, and then stored under dark for 10 h (gray area) under N_2_ environment. The reverse-scan efficiency was recorded as well at 100 mV s^−1^ scan rate after each 10-h MPP operation. The stabilized efficiency at the red point was obtained by replacing the degraded Spiro-OMeTAD layer with a fresh one. **c** *J*-*V* curves of the control device and PSG device before MPP tests and after MPP operation of 300 s for the control device and after MPP operation of 200 h for the PSG device

As shown in Fig. 6a, in the absence of Cs incorporation (for both control and non-Cs PSG devices), the steady-state output of FAPbI_3_-based perovskite solar cells exhibits an obvious drop in the first 300 s operation under AM1.5 illumination. In contrast, stable output is achieved in the case of the Cs-containing PSG devices. We further examine the long-term operational stability of Cs-containing PSG devices operating at MPP under one sun illumination (Fig. 6b). These devices were operated at 1 sun MPP for about 10 h, and then stored under dark for about 10 h, and then this cycle was repeated, with the goal of emulating solar cell working conditions^27,46^. At the end of each continuous MPP tracking, a reverse-scan efficiency was recorded as well. The device retains 60% of initial stabilized efficiency after 140 h MPP operation (corresponding to 280 h stability test) when direct MPP tracking is employed. It should be noted that there is a discrepancy between steady-state and reverse-scan PCEs after 20 h MPP operation. This highlights the necessity of using MPP tracking, instead of taking the reverse *J*-*V* scans for long-term operational stability tests, a point discussed recently by W. Tress et al^27^.

We further compare the *J*-*V* curves of the control and PSG devices before and after degradation (Fig. 6c). The main efficiency loss in Cs-containing PSG devices after 200-h MPP operation (from 20.7 to 13.8%) and control device after 300-s MPP operation (from 18.7 to 10.9%) originates from losses in *J*_sc_ and fill factor. The *J*_sc_ loss in Cs-containing PSG devices arises mainly from deterioration in the Spiro-OMeTAD layer^48,52^: when we replace the Spiro-OMeTAD with a fresh layer (Supplementary Fig. 17), the *J*_sc_ value recovers to 23.6 mA cm^−2^ and the stabilized efficiency recovers to 80% of the initial value (red dot in Fig. 6b). Thermal stability is another concern in solar applications due to illumination-induced heating. We examine the accelerated thermal decomposition on the perovskite films with and without Cs. The decomposition of perovskite film is strikingly suppressed when Cs-containing perovskite seeds are used (Supplementary Fig. 17).

## Discussion

In summary, we report a PSG approach to fabricate high-quality, Cs-containing FAPbI_3_-based perovskite films with a bandgap below 1.55 eV. The perovskite films with seed-assisted growth exhibit large grain size and preferential crystallographic orientation. The perovskites embedded in the PbI_2_ film serve as seed crystals that facilitate perovskite growth. Using Cs-containing perovskite seeds, we successfully incorporate Cs cations into FAPbI_3_-based perovskite films. This PSG approach enables us to fabricate high-performance planar perovskite solar cells with significantly enhanced efficiency and operational stability. Further efficiency and stability improvements are expected via advanced defect engineering (e.g., iodide management and interfacial defect passivation) and by using stable hole-transport layer^2,19,24^.

## Methods

### Device fabrication

Pre-patterned indium tin oxide (ITO)-coated glass was sequentially cleaned using detergent, acetone, and IPA. TiO_2_-Cl nanocrystal solutions were synthesized based on previous work^6^. The TiO_2_-Cl films (50 nm in thickness) were spin-coated on the ITO substrates at 3000 rpm for 30 s and were then annealed on a hot plate at 150 °C for 30 min in ambient air. The substrates were immediately transferred to the N_2_-filled glovebox after cooling. To make a PbI_2_ solution (1.4 M), 760 mg PbI_2_ was dissolved in 1 mL DMF and 160 μL DMSO. The clear PbI_2_ solution was obtained by stirring at 70 °C for 12 h. For PSG, the perovskite seed solution (1.4 M) was first prepared in 2 mL mixed solvent (DMF/DMSO = 4:1 volume ratio) with different compositions as listed in Supplementary Table 3.

The perovskite seed solution was stirred at 50 °C for 30 min. The PbI_2_/perovskite seed mixed solution was obtained by adding perovskite colloidal solution into the PbI_2_ solution. To make FAI/MABr/MACl solution, 1100 mg FAI, 110 mg MABr, and 115 mg MACl were dissolved in 15 mL iso-propanol. The perovskite films were fabricated by two-step sequential deposition. First, the PbI_2_ solution or PbI_2_/perovskite seed mixed solution was spin-coated on ITO/TiO_2_-Cl substrates at 1600 rpm for 20 s and 4000 rpm for 50 s. After spin coating, the film was annealed at 70 °C for 2 min. The as-prepared FAI/MABr/MACl solution was subsequently spin-coated on the PbI_2_ or PbI_2_/seed layer at 2000 rpm for 20 s. The film was then annealed at 140 °C for 25 min in air with 25% RH. After cooling down to room temperature, the perovskite film was washed again by drop-casting IPA on the surface. The hole-transport layer (Spiro-OMeTAD) was deposited on perovskite films by spin coating a solution (chlorobenzene solvent) containing 65 mg mL^−1^ Spiro-OMeTAD, 70 μL mL^−1^ bis(trifluoromethane)sulfonimide lithium salt (170 mg mL^−1^ in acetonitrile), 20 μL mL^−1^ tert-butylpyridine, at the rate of 40,000 rpm for 30 s. Spiro-OMeTAD is bought from Xi'an Baolaite Photoelectric Technology Co., Ltd. Finally, 100 nm Au contact was deposited on top of Spiro-OMeTAD by electron-beam evaporation in an Angstrom Engineering deposition system.

### Solar cell characterization

Current density-voltage (*J*-*V*) curves were obtained by using a Keithley 2400 current-voltage meter under AM1.5G illumination (Newport, Class A). Unless otherwise stated, *J*-*V* curves were obtained by using a scanning rate of 100 mV s^−1^ from 1.15 to −0.01 V with 10 mV voltage step. The steady-state efficiency was measured by setting the bias voltage to the initial *V*_MPP_ and then tracing the current density. To calculate the photocurrent density, we define the area using an aperture (0.049 cm^2^) placed on the glass side of device to avoid overestimation of the photocurrent density. EQE measurements were performed using a Newport system (QuantX-300) calibrated by a certified silicon solar cells without any bias light. The stability tests were performed at continuous MPP under AM 1.5G one-sun illumination with a 420 nm long-pass UV-filter under N_2_ environment (equivalent to white LED illumination used in other reports). Without filtering the UV illumination from the solar simulator, the efficiency drops faster and irreversibly possibly due to the interface degradation related to TiO_2_ (Supplementary Fig. 18)^53,54^. The cells were purged with nitrogen flow for 1 h in the testing box before MPP tracking to get rid of residual moisture on the surface. The continuous current output was then recorded. No excess encapsulation and preconditioning procedure is used.

### Optical characterization

PL was measured using a Horiba Fluorolog time correlated single-photon-counting system with photomultiplier tube detectors. The light was illuminated from the glass side of the perovskite film. The excitation source is a laser diode at a wavelength of 540 nm. In situ wide-field PL imaging was carried out on a home-built inverted optical microscope (Ti-U, Nikon). An air objective (0.92 NA, 60× magnification) was used. A 488 nm laser line from an argon laser was used as the excitation light. A pair of half-wave and quarter-wave plates were used for converting the linear polarized laser light into the circular-polarized light. A lens was used to expand the laser beam for wide-field excitation. The PL emission collected by the same objective was filtered with a 500 nm long-pass filter before reaching an EMCCD camera (Hamamatsu). The PbI_2_/perovskite film was prepared onto a clean glass cover slide (170 μm in thickness) instead of the thick glass substrate often used for solar cells. FAI/MABr/MACl solution in anhydrous IPA was then dropped on to the film while PL imaging started. PL video was acquired with a frame rate of 50 ms. PL spectral mapping was carried out with 488 nm laser excitation using a home-built confocal microscope on a 20 × 20 μm^2^ sample area with a resolution of 100 × 100 pixels. The growth rate of perovskite is obtained by measuring the diameter change of red spot (indicating perovskite) after 5 s reaction. Dynamic light scattering was conducted on DelsaMax PRO light scattering analyzer. A 50 mW diode-pumped solid-state single longitudinal mode laser was used and operated at 532 nm. The DelsaMax Analysis software was used to analyze the light scattering data obtained using the QELS function to determine a hydrodynamic radius. The tube was washed for three times by DMF before injecting the solution.

### Additional materials characterization

High-resolution SEM images were obtained using the Hitachi S-5200 microscope with an accelerating voltage of 1 kV. XRD patterns were collected using a Rigaku MiniFlex 600 diffractometer equipped with a NaI scintillation counter and using monochromatized Copper Kα radiation (*λ* = 1.5406 Å). XPS analysis was carried out using the Thermo Scientific K-Alpha XPS system, with a 300 μm spot size, 75 eV pass energy, and energy steps of 0.05 eV. Perovskite thin films were prepared on ITO substrates and electron flood gun was used for charge compensation to avoid peak shifting. All signals were normalized to the Pb signals for direct comparison between different samples. Optical absorption measurements were carried out in a Lambda 950 UV/Vis spectrophotometer. The impedance spectrum was measured using a potentiostat electrochemical workstation (AUT50690, PGSTAT204, The Netherlands) at different biases. The frequency ranges from 1 MHz to 0.01 Hz with 100 data points. The characteristic relaxation time is obtained by using R_rec_C_rec_ (see equivalent circuit in Fig. S14). The Warburg impedance (*W*_s_) is added to the equivalent circuit due to ion migration in perovskite, which is manifested as a semi-infinite circle at the low-frequency part (below 10 Hz). The defect density is estimated from the frequency-dependent capacitance using the following equation:

1 $$N_{\mathrm{t}}(f) = - (V_{{\mathrm {bi}}} - V_{{\mathrm {app}}})/q{ {WkT}} \times ({\mathrm {d}}C/{\mathrm {d}}f) \times f,$$

where *V*_bi_, *W*, and *V*_app_ stand for the build-in voltage, the width of the space charge region, and applied voltage, respectively. The frequency is converted from frequency to energy level of defects (*E*_a_ = *E*_t_ − *E*_c_/*E*_v_) using thermal admittance spectroscopy. Then we derived the defect distribution under different bias voltages as a function of energy level:

2 $$N_{\mathrm{t}}(E_{\mathrm {a}}) = - (V_{{\mathrm {bi}}} - V_{{\mathrm {app}}})/q{ {WkT}} \times ({\mathrm {d}}C/{\mathrm {d}}f) \times f.$$

To identify the energy levels of defects, we adopted used the method to link frequency and energy level in related prior literature^47^.

ToF-SIMS measurements were carried out with a time-of-flight secondary ion mass spectrometer (TOF.SIMS 5) from IONTOF company. Ar-ion is used to etch the perovskite layer-by-layer and then the Bi ion is used to create segments with positive or negative charge. The sputtering rate is 0.22 nm s^−1^ for SiO_2_ with 10 KeV energy under GCIB mode. The polarity of ionic beam was kept positive.

### Data availability

Data that support the findings of this study are available in separate Supplementary Data Files in Supplementary Information section. All other relevant data are available from the corresponding authors upon reasonable request.

## Electronic supplementary material

Supplementary Information

Peer Review File

Description of Additional Supplementary Files

Supplementary Data 1

Supplementary Data 2

Supplementary Data 3

Supplementary Data 4

Supplementary Data 5

Supplementary Data 6

Supplementary Data 7

Supplementary Data 8

Supplementary Data 9

## References

[1] Gratzel M (2014). The light and shade of perovskite solar cells. Nat. Mater..

[2] Yang WS (2017). Iodide management in formamidinium-lead-halide-based perovskite layers for efficient solar cells. Science.

[3] Yang WS (2015). High-performance photovoltaic perovskite layers fabricated through intramolecular exchange. Science.

[4] Bi D (2016). Polymer-templated nucleation and crystal growth of perovskite films for solar cells with efficiency greater than 21%. Nat. Energy.

[5] Brenner TM, Egger DA, Kronik L, Hodes G, Cahen D (2016). Hybrid organic–inorganic perovskites: low-cost semiconductors with intriguing charge-transport properties. Nat. Rev. Mater..

[6] Tan H (2017). Efficient and stable solution-processed planar perovskite solar cells via contact passivation. Science.

[7] Jeon NJ (2014). Solvent engineering for high-performance inorganic-organic hybrid perovskite solar cells. Nat. Mater..

[8] Ummadisingu A (2017). The effect of illumination on the formation of metal halide perovskite films. Nature.

[9] Burschka J (2013). Sequential deposition as a route to high-performance perovskite-sensitized solar cells. Nature.

[10] Jiang Q (2016). Enhanced electron extraction using SnO_2_ for high-efficiency planar-structure HC(NH2)2PbI3-based perovskite solar cells. Nat. Energy.

[11] Protesescu L (2015). Nanocrystals of cesium lead halide perovskites (CsPbX(3), X=Cl, Br, and I): novel optoelectronic materials showing bright emission with wide color gamut. Nano. Lett..

[12] Jeon NJ (2015). Compositional engineering of perovskite materials for high-performance solar cells. Nature.

[13] Eperon GE (2014). Formamidinium lead trihalide: a broadly tunable perovskite for efficient planar heterojunction solar cells. Energy Environ. Sci..

[14] Saliba M (2016). Cesium-containing triple cation perovskite solar cells: improved stability, reproducibility and high efficiency. Energy Environ. Sci..

[15] Saliba M (2016). Incorporation of rubidium cations into perovskite solar cells improves photovoltaic performance. Science.

[16] McMeekin DP (2016). A mixed-cation lead mixed-halide perovskite absorber for tandem solar cells. Science.

[17] Zhou W (2017). Light-independent ionic transport in inorganic perovskite and ultrastable Cs-based perovskite solar cells. J. Phys. Chem. Lett..

[18] Fang H (2015). Photoexcitation dynamics in solution-processed formamidinium lead iodide perovskite thin films for solar cell applications. Light Sci. Appl..

[19] Zheng X (2017). Defect passivation in hybrid perovskite solar cells using quaternary ammonium halide anions and cations. Nat. Energy.

[20] Bush KA (2017). 23.6%-efficient monolithic perovskite/silicon tandem solar cells with improved stability. Nat. Energy.

[21] Green MA (2018). Solar cell efficiency tables (version 51). Prog. Photovolt. Res. Appl..

[22] Zhou, N. et al. CsI pre-intercalation in the inorganic framework for efficient and stable FA1-x Csx PbI3 (Cl) perovskite solar cells. *Small* **13**, 1700484 (2017). 10.1002/smll.201700484 28464500

[23] Yang D (2016). Surface optimization to eliminate hysteresis for record efficiency planar perovskite solar cells. Energy Environ. Sci..

[24] Arora N (2017). Perovskite solar cells with CuSCN hole extraction layers yield stabilized efficiencies greater than 20%. Science.

[25] Wang, Z. et al. Efficient and air-stable mixed-cation lead mixed-halide perovskite solar cells with n-doped organic electron extraction layers. *Adv. Mater*. **29**, 1604186 (2017). 10.1002/adma.201604186 27905138

[26] Wang Z (2017). Efficient ambient-air-stable solar cells with 2D–3D heterostructured butylammonium-caesium-formamidinium lead halide perovskites. Nat. Energy.

[27] Domanski K, Alharbi EA, Hagfeldt A, Grätzel M, Tress W (2018). Systematic investigation of the impact of operation conditions on the degradation behaviour of perovskite solar cells. Nat. Energy.

[28] Kulbak M, Cahen D, Hodes G (2015). How important is the organic part of lead halide perovskite photovoltaic cells? Efficient CsPbBr3 cells. J. Phys. Chem. Lett..

[29] Beal RE (2016). Cesium lead halide perovskites with improved stability for tandem solar cells. J. Phys. Chem. Lett..

[30] Eperon GE (2015). Inorganic caesium lead iodide perovskite solar cells. J. Mater. Chem. A.

[31] Sharenko A, Toney MF (2016). Relationships between lead halide perovskite thin-film fabrication, morphology, and performance in solar cells. J. Am. Chem. Soc..

[32] Bridgman PW (1925). Certain physical properties of single crystals of tungsten, antimony, bismuth, tellurium, cadmium, zinc, and tin. Proc. Am. Acad. Arts Sci..

[33] Fisher G, Seacrist MR, Standley RW (2012). Silicon crystal growth and wafer technologies. Proc. IEEE.

[34] Chen Q, Jiang Y, Yan J, Qin M (2008). Progress in modeling of fluid flows in crystal growth processes. Prog. Nat. Sci..

[35] Habisreutinger SN, McMeekin DP, Snaith HJ, Nicholas RJ (2016). Research update: strategies for improving the stability of perovskite solar cells. APL Mater..

[36] Lee JW (2015). Formamidinium and cesium hybridization for photo- and moisture-stable perovskite solar cell. Adv. Energy Mater..

[37] Yu Y (2016). Improving the performance of formamidinium and cesium lead triiodide perovskite solar cells using lead thiocyanate additives. ChemSusChem.

[38] Bi C (2015). Non-wetting surface-driven high-aspect-ratio crystalline grain growth for efficient hybrid perovskite solar cells. Nat. Commun..

[39] Ahmad S, Kanaujia PK, Niu W, Baumberg JJ, Vijaya Prakash G (2014). In situ intercalation dynamics in inorganic-organic layered perovskite thin films. ACS Appl. Mater. Interfaces.

[40] Yan K (2015). Hybrid halide perovskite solar cell precursors: colloidal chemistry and coordination engineering behind device processing for high efficiency. J. Am. Chem. Soc..

[41] Cao J (2016). Identifying the molecular structures of intermediates for optimizing the fabrication of high-quality perovskite films. J. Am. Chem. Soc..

[42] Nayak PK (2016). Mechanism for rapid growth of organic-inorganic halide perovskite crystals. Nat. Commun..

[43] Wong AB (2015). Growth and anion exchange conversion of CH3NH3PbX3Nanorod arrays for light-emitting diodes. Nano. Lett..

[44] Yuan Y, Huang J (2016). Ion migration in organometal trihalide perovskite and its impact on photovoltaic efficiency and stability. Acc. Chem. Res..

[45] Shao, Y., Xiao, Z., Bi, C., Yuan, Y., Huang, J. Origin and elimination of photocurrent hysteresis by fullerene passivation in CH_3_NH_3_PbI_3_ planar heterojunction solar cells. *Nat. Commun*. **5**, 5784 (2014). 10.1038/ncomms6784 25503258

[46] Zhao Y (2016). Correlations between immobilizing ions and suppressing hysteresis in perovskite solar cells. ACS Energy Lett..

[47] Duan HS (2015). The identification and characterization of defect states in hybrid organic-inorganic perovskite photovoltaics. Phys. Chem. Chem. Phys..

[48] Zhao Y (2017). Mobile-ion-induced degradation of organic hole-selective layers in perovskite solar cells. J. Phys. Chem. C.

[49] Dong Q (2014). Electron-hole diffusion lengths 175 mm in solution-grown CH_3_NH_3_PbI_3_ single crystal. Science.

[50] Noel NK (2014). Enhanced photoluminescence and solar cell performance via lewis base passivation of organic inorganic lead halide perovskites. ACS Nano.

[51] Zhao Y (2016). Quantification of light-enhanced ionic transport in lead iodide perovskite thin films and its solar cell applications. Light Sci. Appl..

[52] Guerrero A (2016). Interfacial degradation of planar lead halide perovskite solar cells. ACS Nano.

[53] Leijtens, T. et al. Overcoming ultraviolet light instability of sensitized TiO_2_ with meso-superstructured organometal tri-halide perovskite solar cells. *Nat. Commun*. **4**, 2885 (2013). 10.1038/ncomms3885 24301460

[54] Shin SS (2017). Colloidally prepared La-doped BaSnO_3_ electrodes for efficient, photostable perovskite solar cells. Science.

