# Supplementary material for: Perovskite seeding growth of formamidinium-lead-iodide-based perovskites for efficient and stable solar cells
Source: Nat Commun. 2018 Apr 23;9:1607. doi: 10.1038/s41467-018-04029-7 (PMC5913260; doi:10.1038/s41467-018-04029-7)
Supplement: Supplementary file 1 — Supplementary Information [file 41467_2018_4029_MOESM1_ESM.pdf]

Supplementary Information for

Perovskite seeding growth of formamidinium-lead-iodide based perovskites for efficient and stable solar cells

Zhao et al.

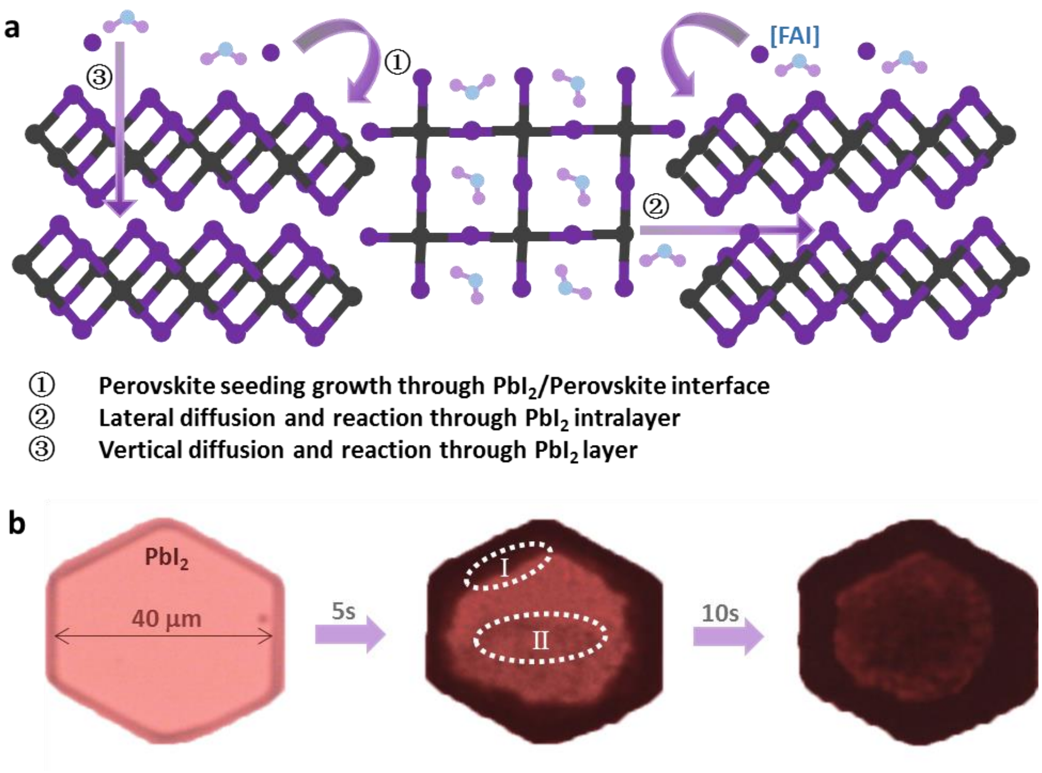

Supplementary Figure 1 | Illustration of efficient intercalation process at PbI_2/perovskite interface. a, Schematic of the reaction dynamics of FAI and PbI_2 in a sequential deposition. Routes 1 and 2 are much faster than route 3. The energy barrier for the crystal growth with seeds is thermodynamically lower than that without seeds. **b,** Optical images of reaction between single crystal PbI_2 and FAI/MABr/MACl solution at different times after dropping the solution. The dashed circle I indicate perovskite/PbI_2 interface and dashed circle II represents the central PbI_2 area. This is followed by a fast growth along the PbI_2/Perovskite interface. Despite some small dots form in the center, they show much slower growth, which may be related to defects or recrystallized PbI_2 clusters at the surface (circle II).

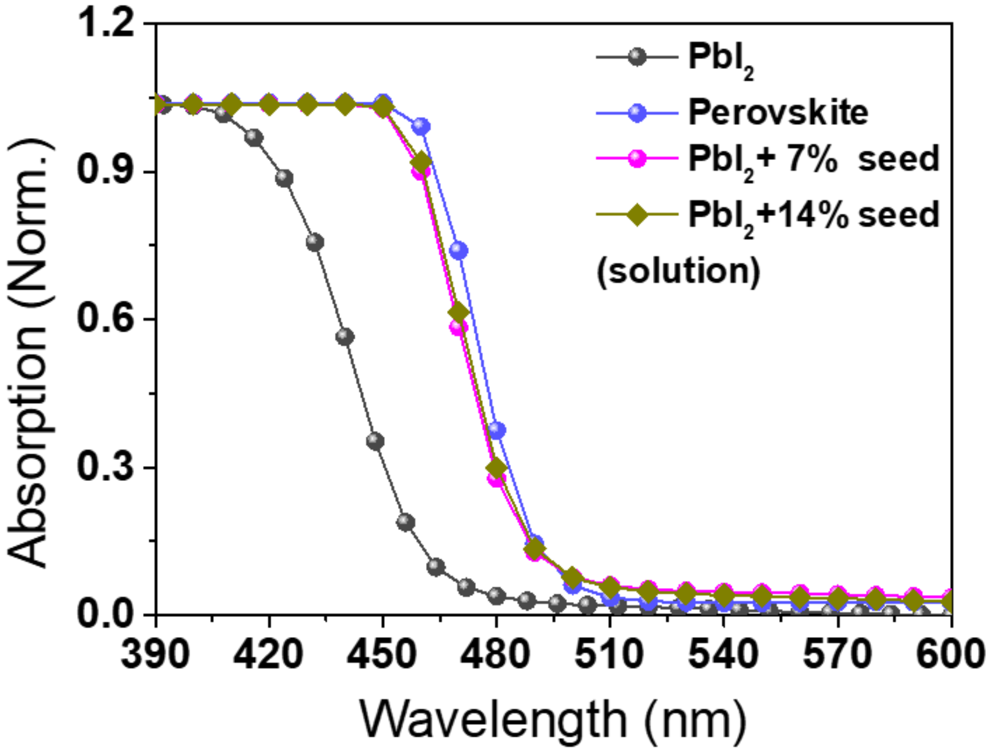

Supplementary Figure 2 | Optical characterization of the colloidal solution. The absorption profile of the PbI_2 solution, perovskite seed solution and the mixed solution of PbI_2 with 7 and 14 vol.% perovskite seed.

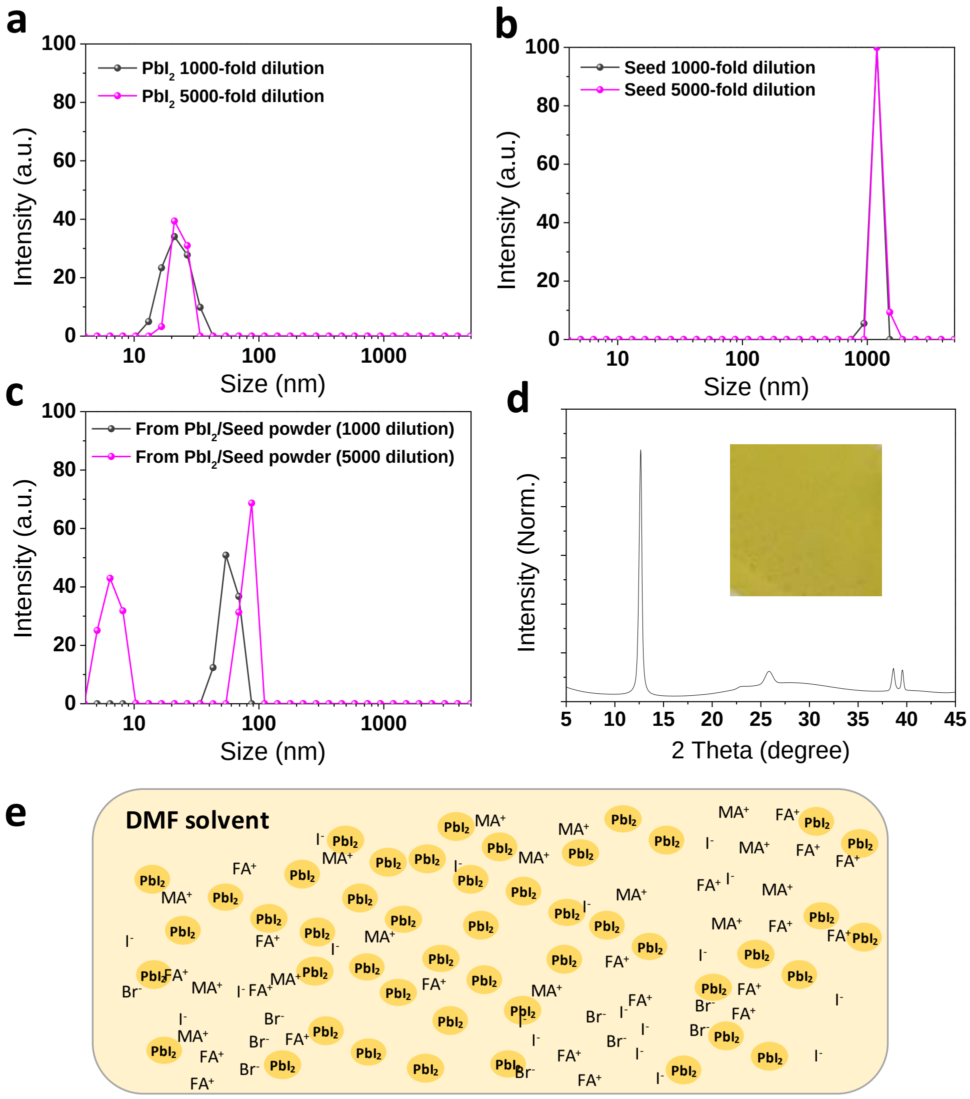

Supplementary Figure 3 丨 a-b, Dynamic light scattering spectra of the pure PbI_2 solution, pure perovskite precursor solution. The as-prepared solution (1.4M) is 1000× and 5000× diluted by DMF, respectively, before measuring. The unchanged spectra indicate a stable existence of these colloids. **c**, Dynamic light scattering of the precursor solution prepared directly from the powder mixture of PbI_2 and perovskite seed crystals (14% molar ratio to PbI_2). The as-prepared solution (1.4M) is 1000× and 5000× diluted by DMF, respectively. The changed spectrum indicate non-stabilized colloids in this solution. **d,** X-ray diffraction pattern of the annealed film prepared from the above solution. Only diffraction peaks of PbI_2 are observed. The inset is the digital photo of the film after annealing. **e,** Schematic view of the distribution of perovskite constitutes in the above solution.

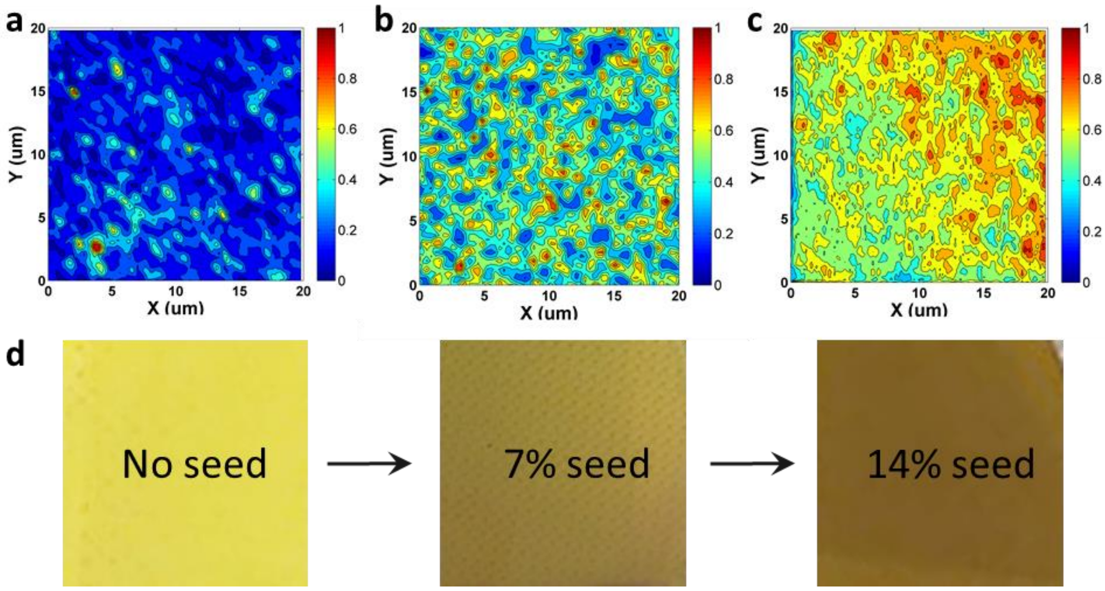

Supplementary Figure 4 | a-c, Confocal PL mapping of the intensity on the PbI_2 films with 7, 20, and 24 vol.% perovskite seed. **d,** Digital photos of the PbI_2 film without seed and with 7 and 14 vol.% perovskite seed.

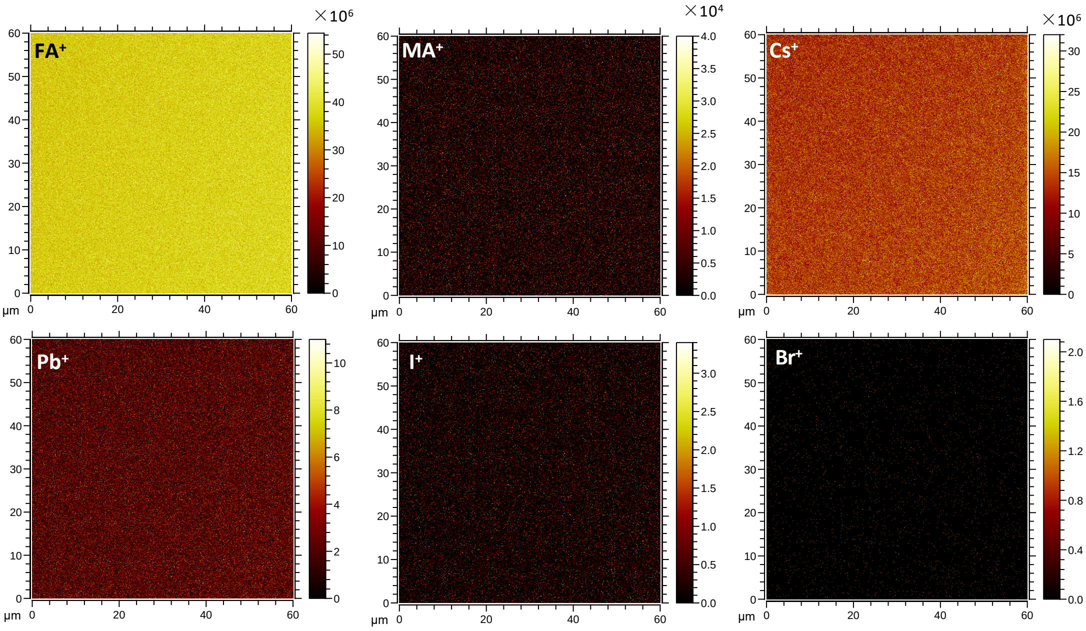

Supplementary Figure 5 | 2-D element mapping of PSG sample after Ar^+ etching for 10 seconds with 10keV ion energy. The element mapping is obtained by dividing 60*60 μm^2 area into 1024*1024 pixels with 60 nm spatial resolution.

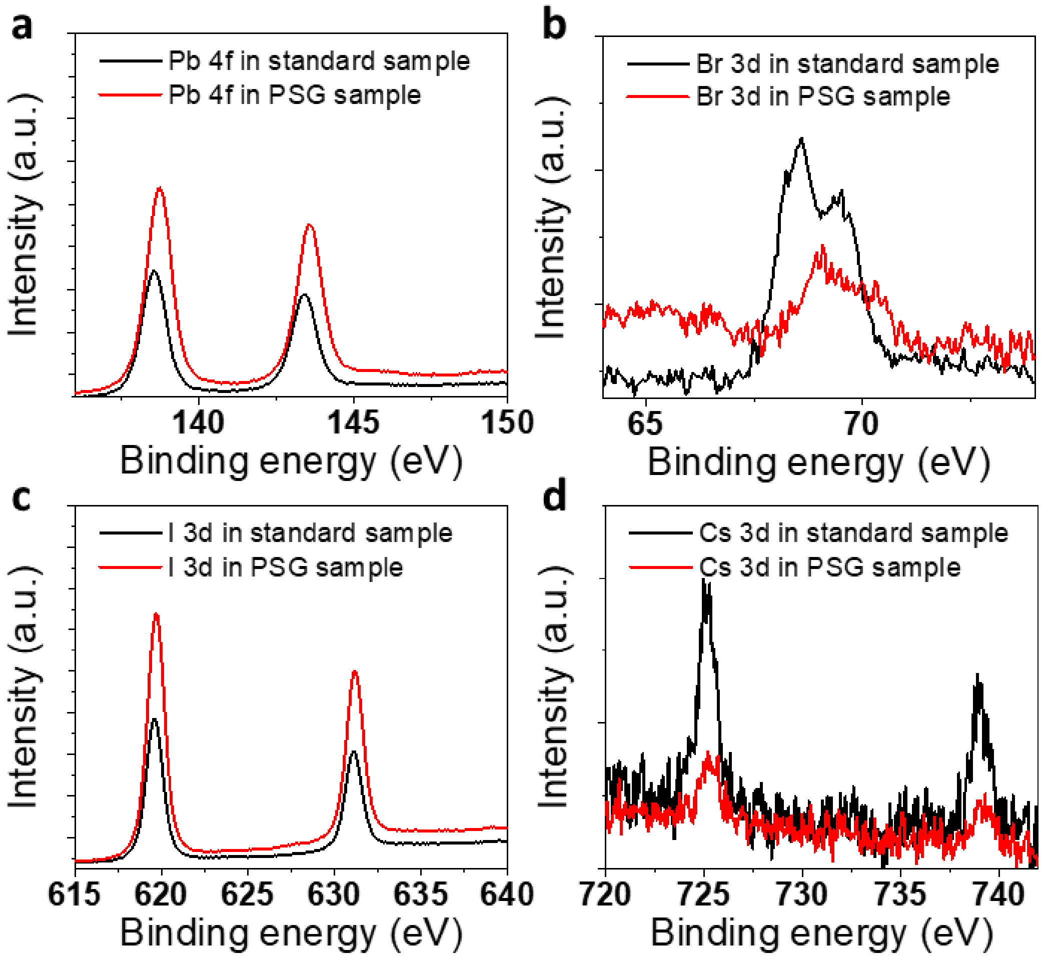

Supplementary Figure 6 | a-d, The X-ray photoelectron spectroscopy for specific element of perovskite films. The sample surface is pre-etched by Ar^+ gun for 10 seconds before measurements. The integrated area represents the ionic ratio in the film. The estimated ratio is further confirmed by comparing it to a standard sample ($Cs_{0.05}FA_{0.81}MA_{0.15}PbI_{2.55}Br_{0.45}$) fabricated via one-step method.

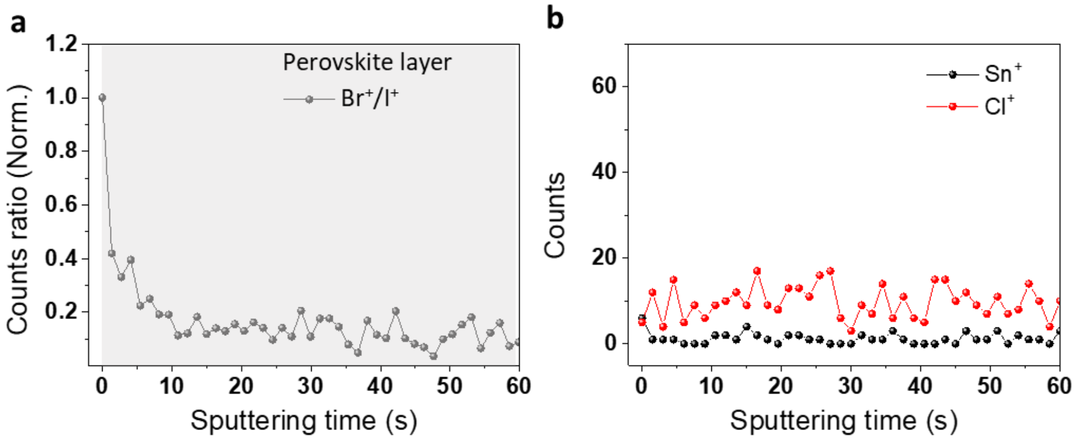

Supplementary Figure 7 | Chemical depth profile of perovskite film. a, The signal counts ratio of bromide to iodide versus Ar^+ sputtering time based on an averaging signal from 60*60 μm^2 area. **b,** The signal from Cl^+ is close to the background noise level represented by Sn^+ signal and is negligible compared to Br or I element.

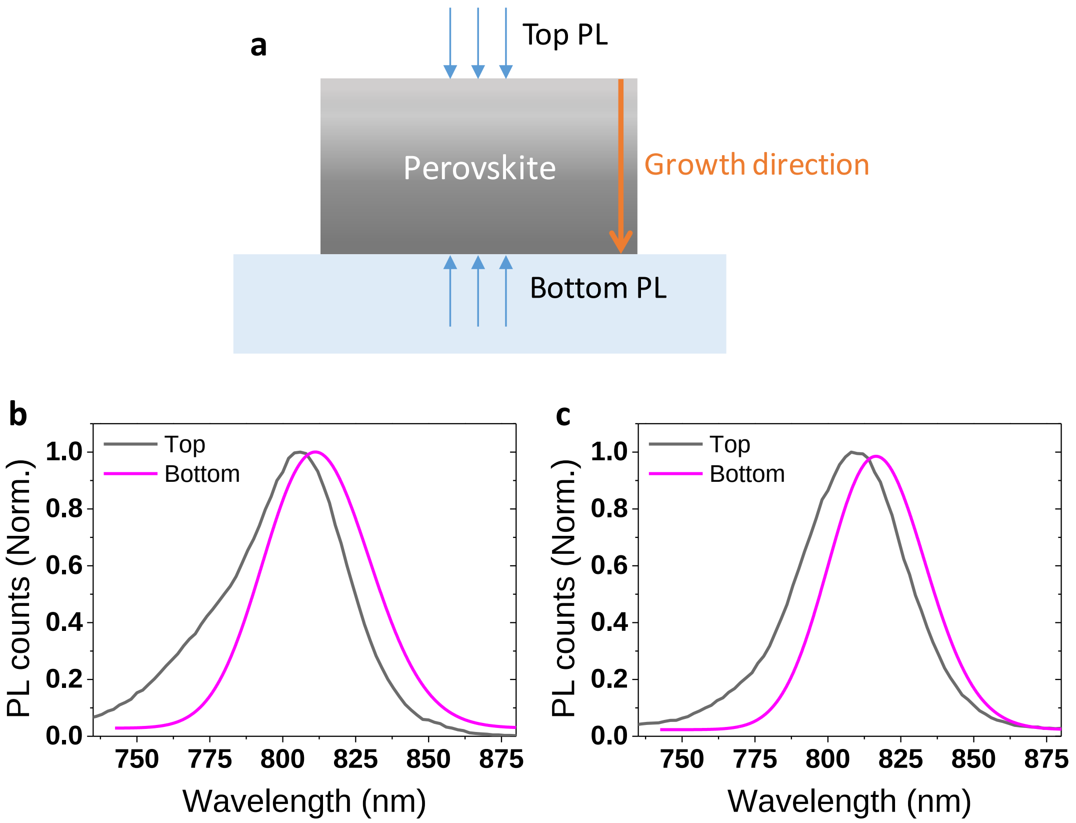

Supplementary Figure 8 | Optical characterization on sequentially-deposited control and PSG perovskite films on glass. a, Schematic of the photoluminescence signal emitted from the top and bottom of the sample. The excitation wavelength is 540 nm. **b-c**, Photoluminescence spectra of control and PSG samples, respectively.

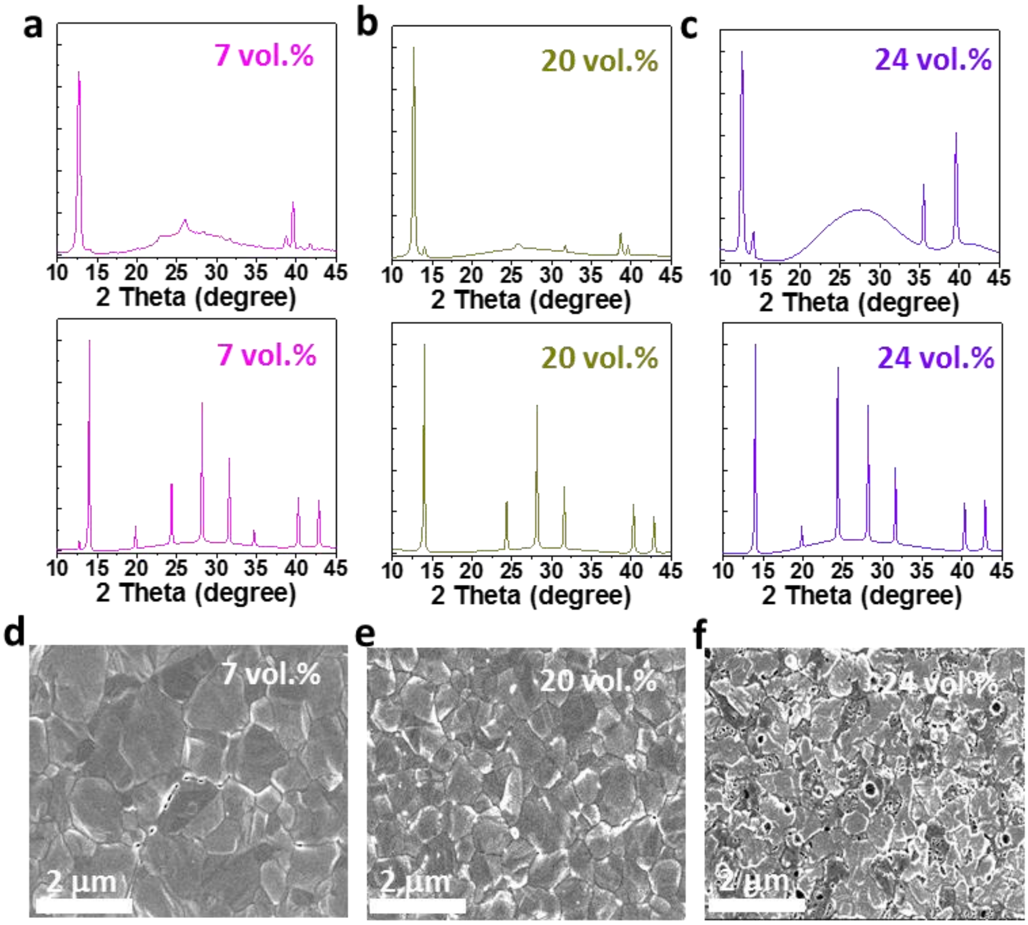

Supplementary Figure 9 | XRD patterns and SEM images of perovskite films prepared with different seed concentrations. a-c. XRD patterns of PbI_2 films with different seed concentrations (upper) and the resulting $FAPbI_3$-based perovskite films (under). **d-f.** SEM images of the perovskite films fabricated by perovskite seeding growth with different seed concentrations.

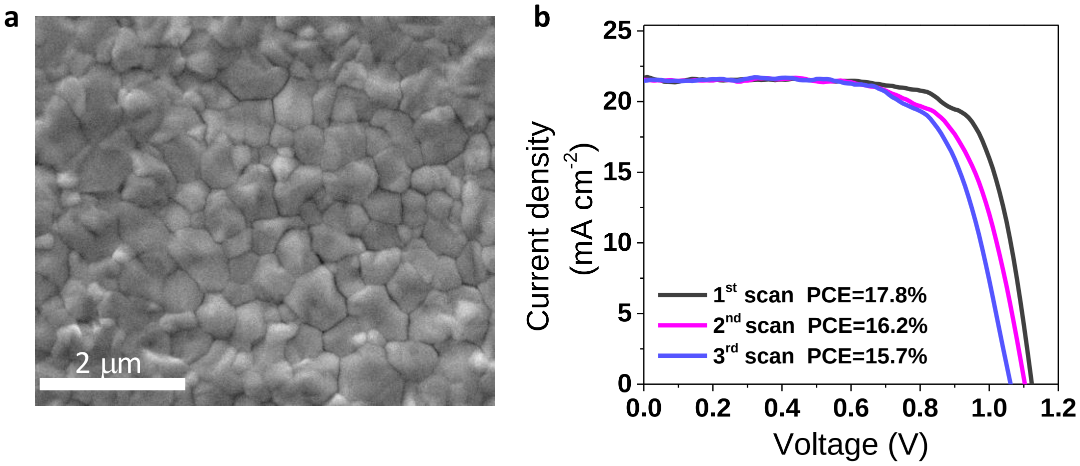

Supplementary Figure 10 | a, SEM images of perovskite films prepared from a PbI_2 film with 14 vol.% $MAPbBr_3$ seed. **b,** The corresponding *J-V* curves for PSG devices fabricated through 14% $MAPbBr_3$ seed, showing low initial efficiency and unstable output.

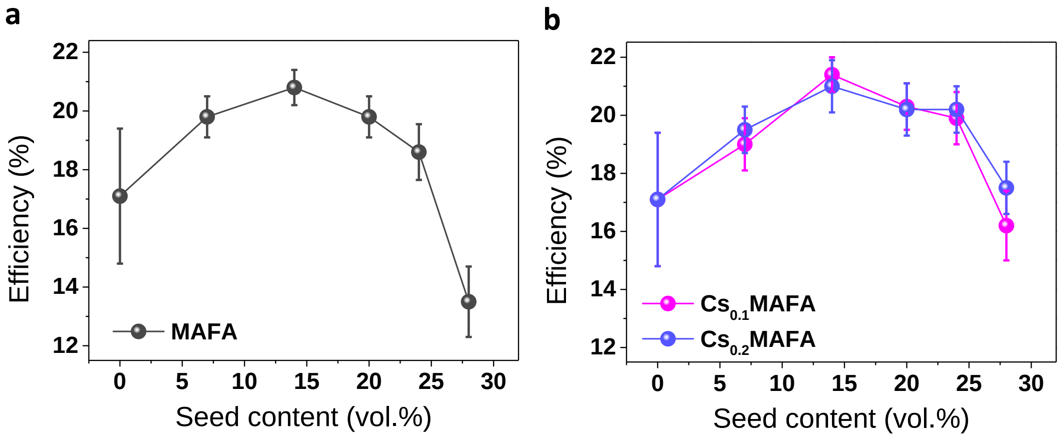

Supplementary Figure 11 | a, The efficiency of perovskite solar cells processed from different seed concentrations by using non-Cs seeds $FA_{0.85}MA_{0.15}PbI_{2.55}Br_{0.45}$ (denoted as MAFA). **b,** The efficiency of perovskite solar cells processed from different seed concentrations by using Cs-containing seeds: $Cs_{0.1}FA_{0.78}MA_{0.12}PbI_{2.55}Br_{0.45}$ (denoted as $Cs_{0.1}$MAFA) and $Cs_{0.2}FA_{0.7}MA_{0.1}PbI_{2.55}Br_{0.45}$ (denoted as $Cs_{0.2}$MAFA). The error bar in above figures represents the discrepancy between the maximum and minimum efficiency value for each condition among at least 24 devices.

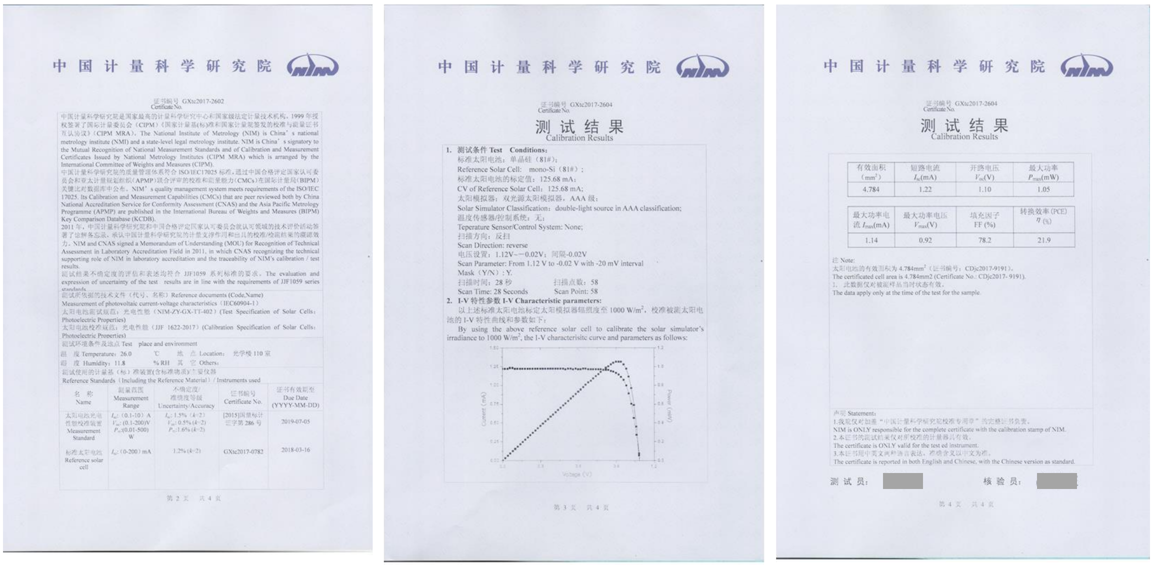

Supplementary Figure 12 | The certified result of PSG device with a mask. The current-voltage scan is performed from 1.12 V to -0.02 V at 40 mV s^{-1} with 20 mV voltage interval. The temperature on the device surface is monitored by a thermocouple, showing 36°C at least. The device has an active area of 0.0487 cm^2 and a PCE of 21.9% (V_{oc}=1.10 V, I_{sc}=1.22 mA, and FF=78.2%).

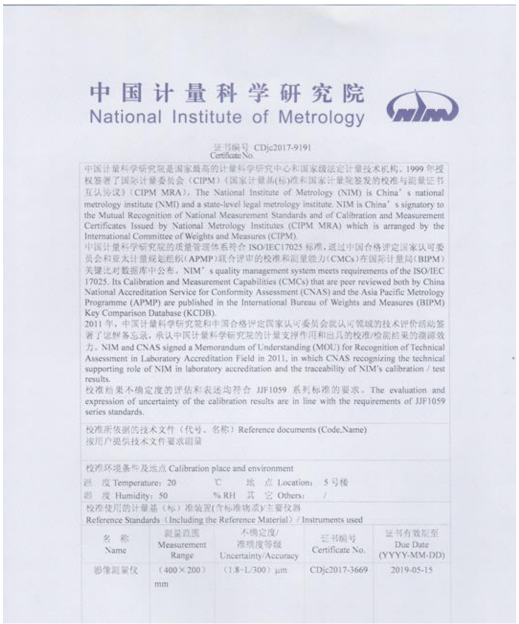

中国计量科学研究院
National Institute of Metrology

证书编号 CDjc2017-9191
Certificate No.

中国计量科学研究院是国家最高的计量科学研究中心和国家级法定计量技术机构，1999 年授权签署了国际计量委员会（CIPM）《国家计量基(标)准和国家计量院签发的校准与测量证书互认协议》（CIPM MRA）。The National Institute of Metrology (NIM) is China's national metrology institute (NMI) and a state-level legal metrology institute. NIM is China's signatory to the Mutual Recognition of National Measurement Standards and of Calibration and Measurement Certificates Issued by National Metrology Institutes (CIPM MRA) which is arranged by the International Committee of Weights and Measures (CIPM).

中国计量科学研究院的质量管理体系符合 ISO/IEC17025 标准，通过中国合格评定国家认可委员会和亚太计量规划组织（APMP）联合评审的校准和测量能力（CMCs）在国际计量局（BIPM）关键比对数据库中公布。NIM's quality management system meets requirements of the ISO/IEC 17025. Its Calibration and Measurement Capabilities (CMCs) that are peer reviewed both by China National Accreditation Service for Conformity Assessment (CNAS) and the Asia Pacific Metrology Programme (APMP) are published in the International Bureau of Weights and Measures (BIPM) Key Comparison Database (KCDB).

2011 年，中国计量科学研究院和中国合格评定国家认可委员会就认可领域的技术评价活动签署了谅解备忘录，承认中国计量科学研究院的计量支撑作用和出具的校准/检测结果的溯源效力。NIM and CNAS signed a Memorandum of Understanding (MOU) for Recognition of Technical Assessment in Laboratory Accreditation Field in 2011, in which CNAS recognizing the technical supporting role of NIM in laboratory accreditation and the traceability of NIM's calibration / test results.

校准结果不确定度的评估和表述均符合 JJF1059 系列标准的要求。The evaluation and expression of uncertainty of the calibration results are in line with the requirements of JJF1059 series standards.

校准所依据的技术文件（代号、名称）Reference documents (Code,Name)
按用户提供技术文件要求测量

校准环境条件及地点 Calibration place and environment
温 度 Temperature：20 ℃ 地 点 Location：5 号楼
湿 度 Humidity：50 %RH 其 它 Others：/

校准使用的计量基（标）准装置(含标准物质)/主要仪器
Reference Standards（Including the Reference Material）/ Instruments used

名 称 Name	测量范围 Measurement Range	不确定度/准确度等级 Uncertainty/Accuracy	证书编号 Certificate No.	证书有效期至 Due Date (YYYY-MM-DD)
影像测量仪	（400×200）mm	（1.8+L/300）μm	CDjc2017-3669	2019-05-15

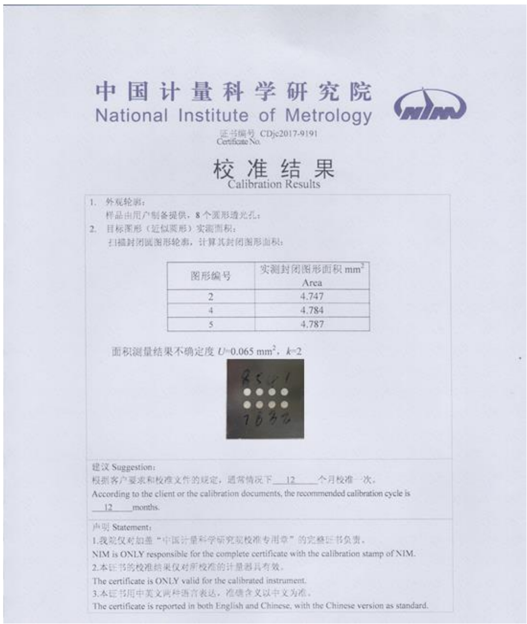

中国计量科学研究院
National Institute of Metrology

证书编号 CDjc2017-9191
Certificate No.

校 准 结 果
Calibration Results

1. 外观轮廓：
样品由用户制备提供，8 个圆形透光孔；
2. 目标图形（近似圆形）实测面积：
扫描封闭圆图形轮廓，计算其封闭图形面积；

图形编号	实测封闭图形面积 mm² Area
2	4.747
4	4.784
5	4.787

面积测量结果不确定度 U=0.065 mm²，k=2

8 5 [illegible] 1
7 6 3 2

建议 Suggestion：
根据客户要求和校准文件的规定，通常情况下 12 个月校准一次。
According to the client or the calibration documents, the recommended calibration cycle is 12 months.

声明 Statement：
1.我院仅对加盖“中国计量科学研究院校准专用章”的完整证书负责。
NIM is ONLY responsible for the complete certificate with the calibration stamp of NIM.
2.本证书的校准结果仅对所校准的计量器具有效。
The certificate is ONLY valid for the calibrated instrument.
3.本证书用中英文两种语言表达，准确含义以中文为准。
The certificate is reported in both English and Chinese, with the Chinese version as standard.

Supplementary Figure 13 丨 The certified area of the mask on a certified device. The uncertainty value is 0.065 mm^2 using optical image analysis.

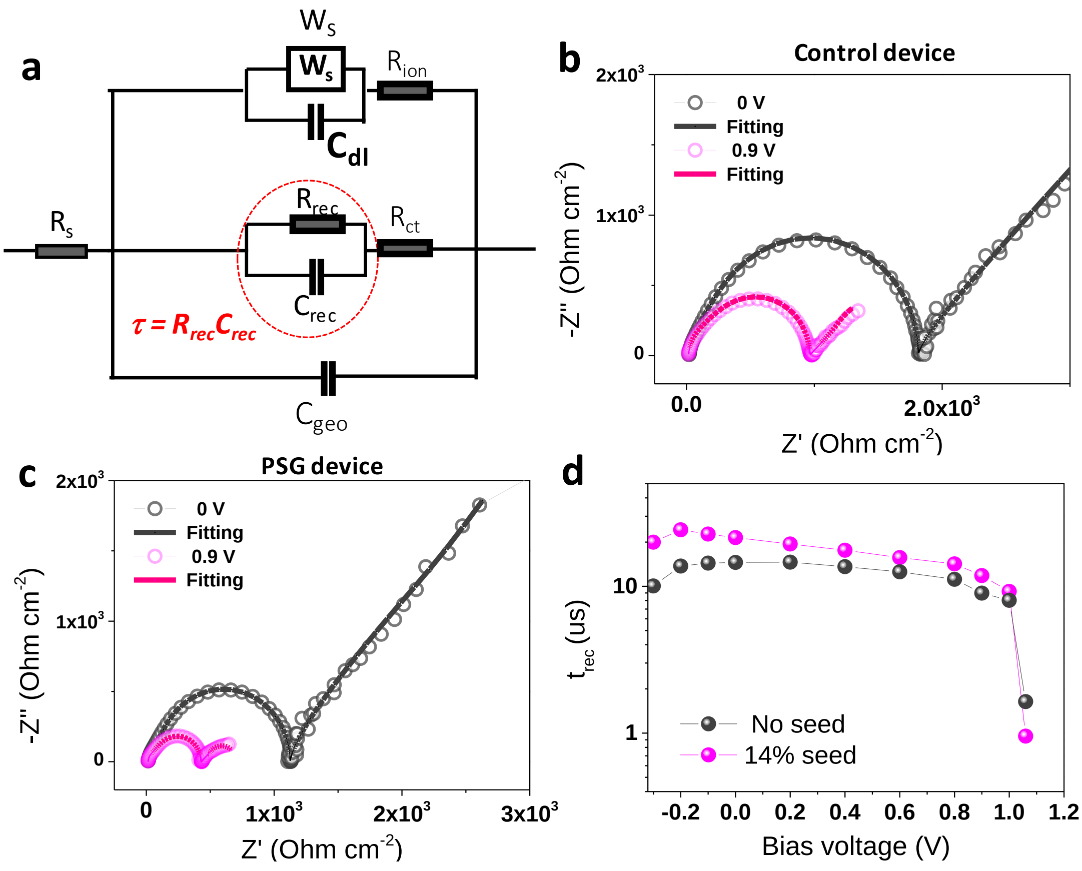

Supplementary Figure 14 | Impedance spectra of perovskite solar cells. a, The equivalent circuit used to fit the impedance spectra. Warburg impedance and the related capacitance are included to fit the low-frequency part. **b,** Impedance spectra of the control solar cell without seed. **c,** Impedance spectra of the PSG solar cell with perovskite seeding. **d,** Time constants of the parasitic recombination ($\tau = R_{rec}C_{rec}$) at different bias voltages for devices with (in pink color) and without (in black color) perovskite seeding growth.

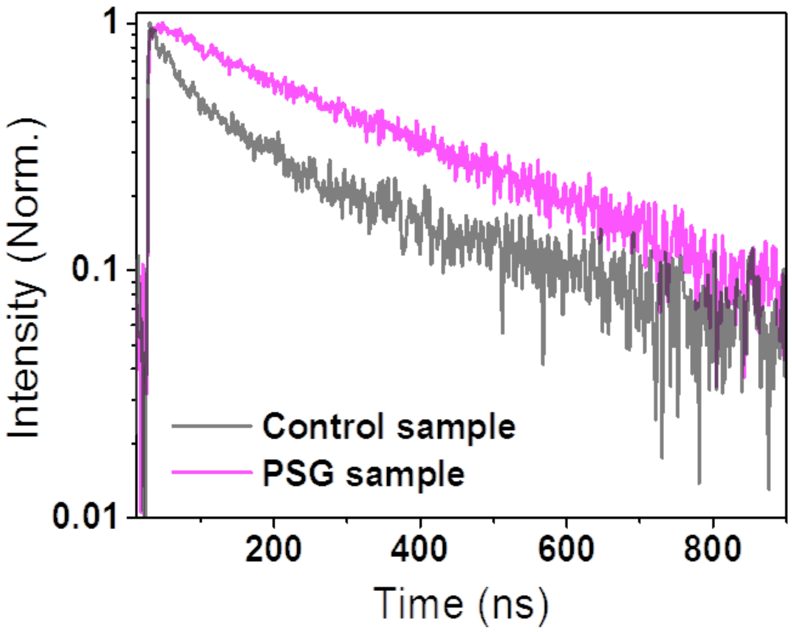

Supplementary Figure 15 | Time-resolved PL decay spectra of control (in gray color) and PSG (in pink color) samples. The signal is collected from the backside of the glass at its peak position with 8 nm collection range.

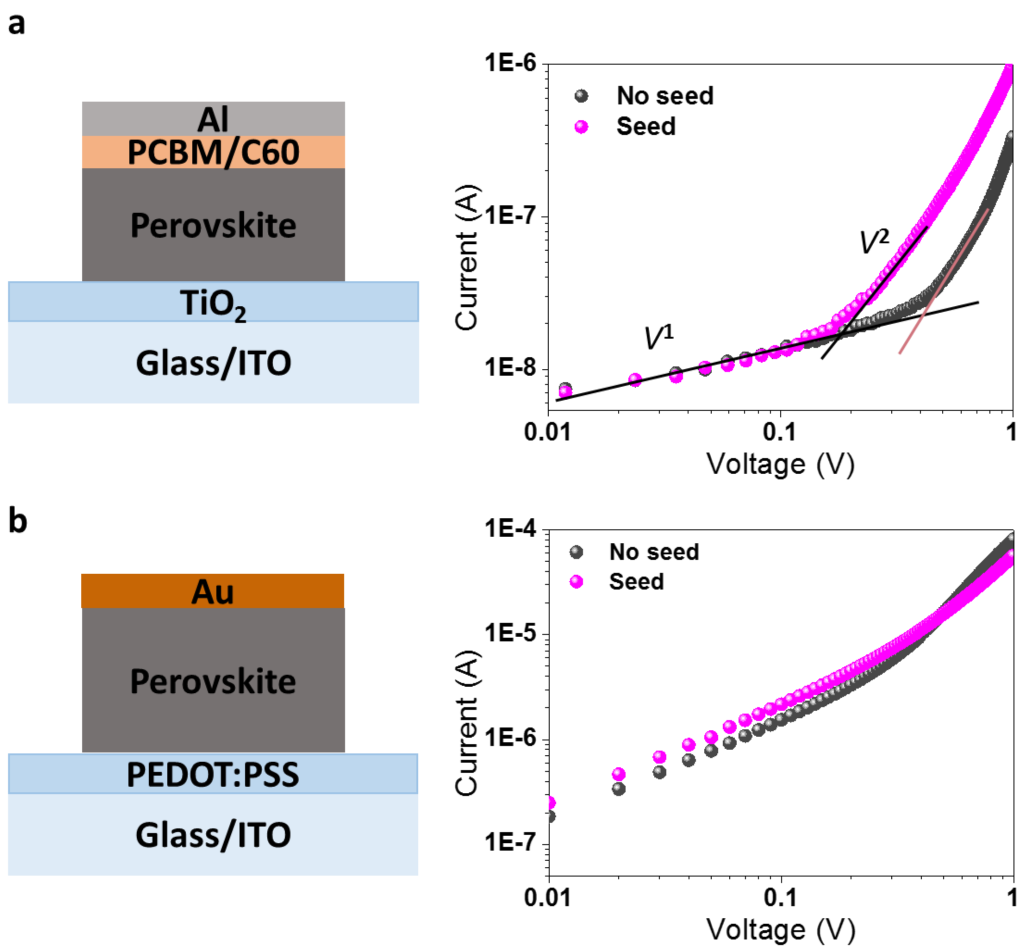

Supplementary Figure 16 | a, Device structure and current-voltage curves for the electron-only devices and **b,** the hole-only devices. The crossover point of two lines with different slopes indicates the trap-filled limited voltage. Lower defects concentration leads to a smaller trap-filled limited voltage.

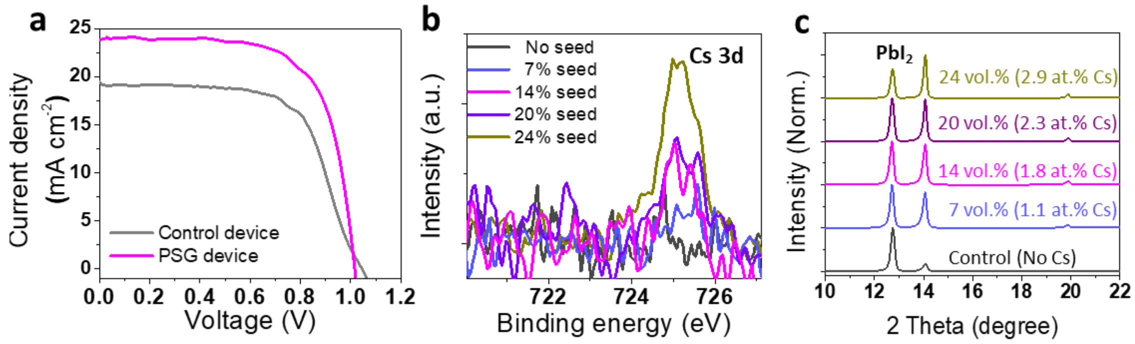

Supplementary Figure 17 | **a,** *J*-*V* curves of the healed control and PSG devices by replacing the degraded Sprio-MeOTAD with a fresh one. **b,** XPS spectra of Cs 3*d* peaks of the resulting perovskite films via perovskite seeding ($Cs_{0.1}$MAFA) with different seed concentrations. The plots are normalized to the intensity of Pb 4*f* peak. **c,** XRD patterns of the perovskite films with different seed concentrations after annealing at 150℃ for 50 mins in the air (25% RH).

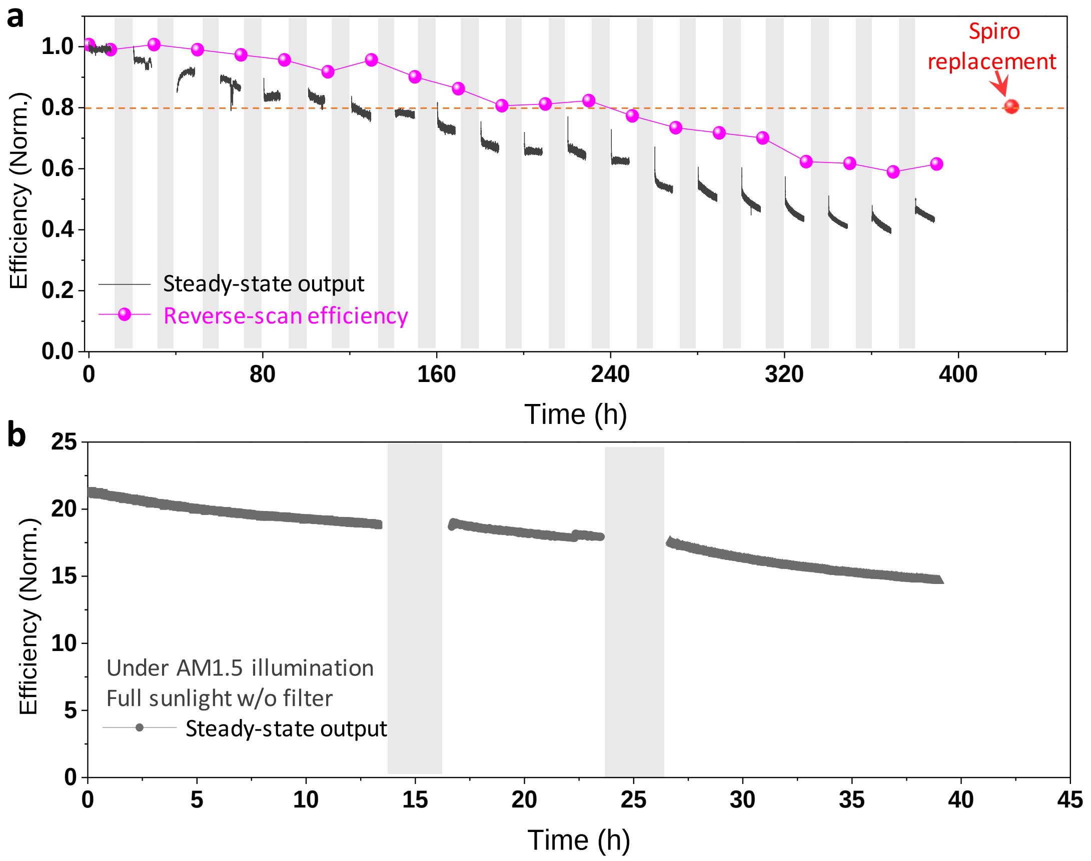

Supplementary Figure 18 | a, Long-term operational stability for the PSG device under AM1.5 illumination with a 420 nm cutoff UV filter. **b,** Steady-state output under AM1.5 illumination without UV filter.

Supplementary Table 1 丨 Summary of operational stability of low bandgap $FAPbI_3$-based perovskite solar cells and higher bandgap, Cs-containing mixed perovskite solar cells.

Material	Composition	Bandgap (eV)	Efficiency (%)	Operational stability under illumination
**Low bandgap, FAPbI3-based perovskites**	$MA_{0.05}FA_{0.95}Pb(I_{0.95}Br_{0.05})_3$	1.53	22.1 (certified)	Not reported (Ref.2)
	$MA_{0.03}FA_{0.97}Pb(I_{0.97}Br_{0.03})_3$	1.53	20.9 (certified)	~10% loss after 3 hours (1 sun illumination only, not MPP operation) (Ref.10)
	$Cs_{0.02}MA_{0.03}FA_{0.95}Pb(I_{0.95}Br_{0.05})_3$	1.53	21.5	Negligible loss in the first 10 hours, 40% loss after 140 hours MPP tracking (1 sun) (Our work)
**Higher bandgap, Cs-containing mixed cation-halide perovskites**	$Cs_{0.05}FA_{0.81}MA_{0.14}PbI_{2.55}Br_{0.45}$	1.60	20.1 (certified)	~10% loss after 500 hours MPP (N_2/1 sun) (Ref.6)
	$Cs_{0.05}(MA_{0.17}FA_{0.83})_{0.95}Pb(I_{0.83}Br_{0.17})_3$	1.61	20.2	~5% loss after 1000 hours MPP (N_2/white LED-light @60℃; CuSCN as HTL) (Ref.24)
	$Rb_{0.05}Cs_{0.05}(MA_{0.17}FA_{0.83})_{0.9}Pb(I_{0.83}Br_{0.17})_3$	1.63		~5% loss after 500 hours MPP (N_2/white LED-light@80℃) (Ref.15)
	$Cs_{0.17}FA_{0.83}Pb(Br_{0.17}I_{0.83})_3$	1.63	14.5	Stable for 1000 hours MPP (Ref.20)
	$Cs_{0.17}FA_{0.83}Pb(I_{0.6}Br_{0.4})_3$ (2-D mixed)	1.72	17.3	20% loss after 1600 hours (under 76 $mWcm^{-2}$ light illumination only, not MPP operation) (Ref.26)

Supplementary Table 2 丨 The photovoltaic performance of PSG and control devices under reverse and forward scans measured at scanning rate of 10 mV s^{-1}.

Device	Scan Direction	V_{oc} (V)	J_{sc} (mA cm^{-2})	FF (%)	PCE (%)
PSG	Reverse	1.13	24.1	81	21.7
	Forward	1.10	24.1	79	21.2
Control	Reverse	1.07	23.7	75	19.4
	Forward	1.05	23.7	69	17.1

Supplementary Table 3 丨 The molecular formula and the weight of each precursor for different perovskite seed solution used in perovskite seeding method (Unit: mg).

Composition	PbI_2	$PbBr_2$	FAI	MABr	CsI
Cs0 ($FA_{0.85}MA_{0.15}PbI_{2.55}Br_{0.45}$)	1104	154.1	409.4	47.1	0
Cs10 ($Cs_{0.1}FA_{0.78}MA_{0.12}PbI_{2.55}Br_{0.45}$)	1104	154.1	371.5	40.3	72.8
Cs20 ($Cs_{0.2}FA_{0.7}MA_{0.1}PbI_{2.55}Br_{0.45}$)	1104	154.1	344	33.6	130
