# Supplementary material for: Perovskite seeding growth of formamidinium-lead-iodide-based perovskites for efficient and stable solar cells
Source: Nat Commun. 2018 Apr 23;9:1607. doi: 10.1038/s41467-018-04029-7 (PMC5913260; doi:10.1038/s41467-018-04029-7)
Supplement: Supplementary file 3 — Description of Additional Supplementary Files [file 41467_2018_4029_MOESM3_ESM.pdf]

Description of Additional Supplementary Files

File Name: Supplementary Data 1
Description: Supplementary Data for absorption spectra

File Name: Supplementary Data 2
Description: Supplementary Data for device IV curve

File Name: Supplementary Data 3
Description: Supplementary Data for DLS DMF solvent_values_all

File Name: Supplementary Data 4
Description: Supplementary Data for DLS PbI2

File Name: Supplementary Data 5
Description: Supplementary Data for DLS perovskite

File Name: Supplementary Data 6
Description: Supplementary Data for EQE

File Name: Supplementary Data 7
Description: Supplementary Data for Impedance spectra with PSG

File Name: Supplementary Data 8
Description: Supplementary Data for Impedance spectra without PSG

File Name: Supplementary Data 9
Description: Supplementary Data for stability test
