# Supplementary material for: Perovskite seeding growth of formamidinium-lead-iodide-based perovskites for efficient and stable solar cells
Source: Nat Commun. 2018 Apr 23;9:1607. doi: 10.1038/s41467-018-04029-7 (PMC5913260; doi:10.1038/s41467-018-04029-7)
Supplement: Supplementary file 2 — Peer Review File [file 41467_2018_4029_MOESM2_ESM.pdf]

Editorial Note: this manuscript has been previously reviewed at another journal that is not operating a transparent peer review scheme. This document only contains reviewer comments and rebuttal letters for versions considered at Nature Communications.

REVIEWERS' COMMENTS:

Reviewer #1 (Remarks to the Author):

The authors have addressed the comments satisfactorily. Recommend for publication.

Reviewer #2 (Remarks to the Author):

The authors have comprehensively addressed the concerns I and the other reviewers had, so I now recommend publication.
